# Surgical amputation of a limb 31,000 years ago in Borneo

**DOI:** 10.1038/s41586-022-05160-8

**Published:** 2022-09-07

**Authors:** Tim Ryan Maloney, India Ella Dilkes-Hall, Melandri Vlok, Adhi Agus Oktaviana, Pindi Setiawan, Andika Arief Drajat Priyatno, Marlon Ririmasse, I. Made Geria, Muslimin A. R. Effendy, Budi Istiawan, Falentinus Triwijaya Atmoko, Shinatria Adhityatama, Ian Moffat, Renaud Joannes-Boyau, Adam Brumm, Maxime Aubert

**Affiliations:** 1 Griffith Centre for Social and Cultural Research, Griffith University, Gold Coast, Queensland Australia; 2 Australian Research Centre for Human Evolution, Griffith University, Nathan, Queensland Australia; 3 Archaeology, School of Social Sciences, University of Western Australia, Crawley, Western Australia Australia; 4 Sydney South East Asian Centre, University of Sydney, Sydney, New South Wales Australia; 5 BRIN, OR Arkeologi, Bahasa dan Sastra, Pusat Riset Arkeometri, Jakarta, Indonesia; 6 School of Humanities, Languages and Social Science, Griffith University, Gold Coast, Queensland Australia; 7 Faculty of Art and Design, Bandung Institute of Technology, Bandung, Indonesia; 8 Balai Pelestarian Cagar Budaya Kalimantan Timur, Samarinda, Indonesia; 9 BRIN, OR Arkeologi, Bahasa dan Sastra, Pusat Riset Lingkungan, Maritim, dan Budaya Berkelanjutan, Jakarta, Indonesia; 10 Archaeology, College of Humanities, Arts and Social Sciences, Flinders University, Bedford Park, South Australia Australia; 11 Geoarchaeology and Archaeometry Research Group (GARG), Southern Cross University, Lismore, New South Wales Australia; 12 Palaeo-Research Institute, University of Johannesburg, Johannesburg, South Africa

**Keywords:** Archaeology, Biological anthropology, Social anthropology

## Abstract

The prevailing view regarding the evolution of medicine is that the emergence of settled agricultural societies around 10,000 years ago (the Neolithic Revolution) gave rise to a host of health problems that had previously been unknown among non-sedentary foraging populations, stimulating the first major innovations in prehistoric medical practices^1,2^. Such changes included the development of more advanced surgical procedures, with the oldest known indication of an ‘operation’ formerly thought to have consisted of the skeletal remains of a European Neolithic farmer (found in Buthiers-Boulancourt, France) whose left forearm had been surgically removed and then partially healed^3^. Dating to around 7,000 years ago, this accepted case of amputation would have required comprehensive knowledge of human anatomy and considerable technical skill, and has thus been viewed as the earliest evidence of a complex medical act^3^. Here, however, we report the discovery of skeletal remains of a young individual from Borneo who had the distal third of their left lower leg surgically amputated, probably as a child, at least 31,000 years ago. The individual survived the procedure and lived for another 6–9 years, before their remains were intentionally buried in Liang Tebo cave, which is located in East Kalimantan, Indonesian Borneo, in a limestone karst area that contains some of the world’s earliest dated rock art^4^. This unexpectedly early evidence of a successful limb amputation suggests that at least some modern human foraging groups in tropical Asia had developed sophisticated medical knowledge and skills long before the Neolithic farming transition.

## Abstract (web summary)

Around 31,000 years ago, a young individual from Borneo had part of their left lower leg surgically amputated, probably as a child, and lived for another 6–9 years after amputation.

## Main

**Fig. 1:**
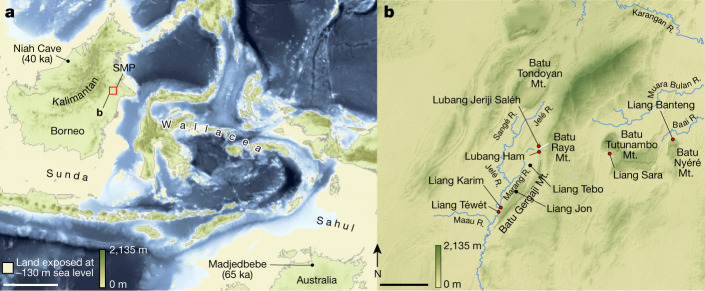
Location of Liang Tebo. **a**, Sunda, the continental shelf region encompassing the present-day island of Borneo during periods of lowered sea levels, is situated to the west of Wallacea and northwest of the Pleistocene low-sea-level landmass of Sahul (Australia and New Guinea). The Sangkulirang–Mangkalihat Peninsula (SMP) is adjacent to the easternmost edge of Sunda. The area shown in **b** is highlighted. **b**, Liang Tebo and surrounding archaeological sites, including those with dated Late Pleistocene rock art (shown in red). Map source, Shuttle Radar Topography Mission 1 Arc-Second Global by NASA/NGS/USGS; GEBCO_2014 Grid, version 20150318 (http://gebco.net). Base maps generated using ArcGIS by M. Kottermair and A. Jalandoni. Scale bars, 500 km (**a**) and 10 km (**b**). ‘ka equals thousands of year’.

**Extended Data Fig. 1:**
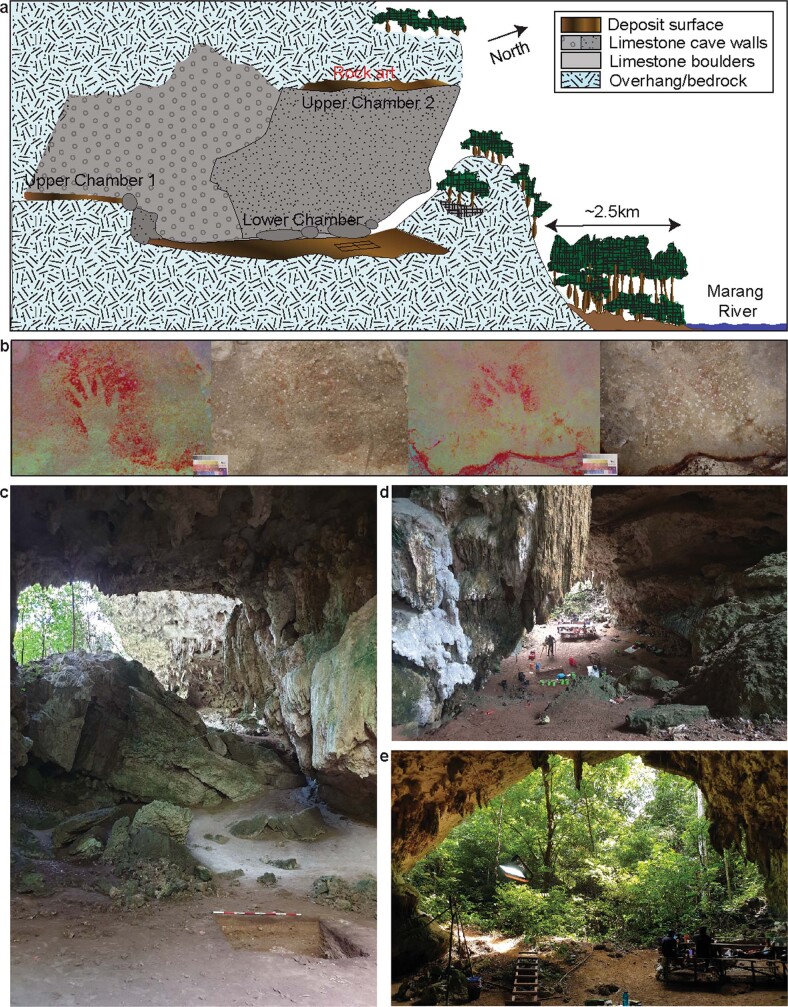
Liang Tebo cave topography and site plan. **a**, Liang Tebo is a cathedral-sized (~160 m^3^) limestone cave with three large chambers. Following GPR and ERT survey, a 2 x 2 m excavation was positioned over an area of likely deep deposit in the centre of the caves’ lower and largest chamber. This area was excavated to a depth of 1.5 m before the project was forced to cease, following the COVID19 pandemic. The human burial (TB1) was excavated in its entirety before the field season was terminated. **b**, The uppermost chamber of the cave contains pigment rock art, including negative hand stencils, shown modified using D-Stretch. **c**, View of excavation square in lower chamber looking south towards upper chamber 1. **d**, View from upper chamber 1 looking northeast into lower chamber. **e**, Liang Tebo north cave mouth showing rainforest vegetation outside of the dripline.

**Extended Data Fig. 2:**
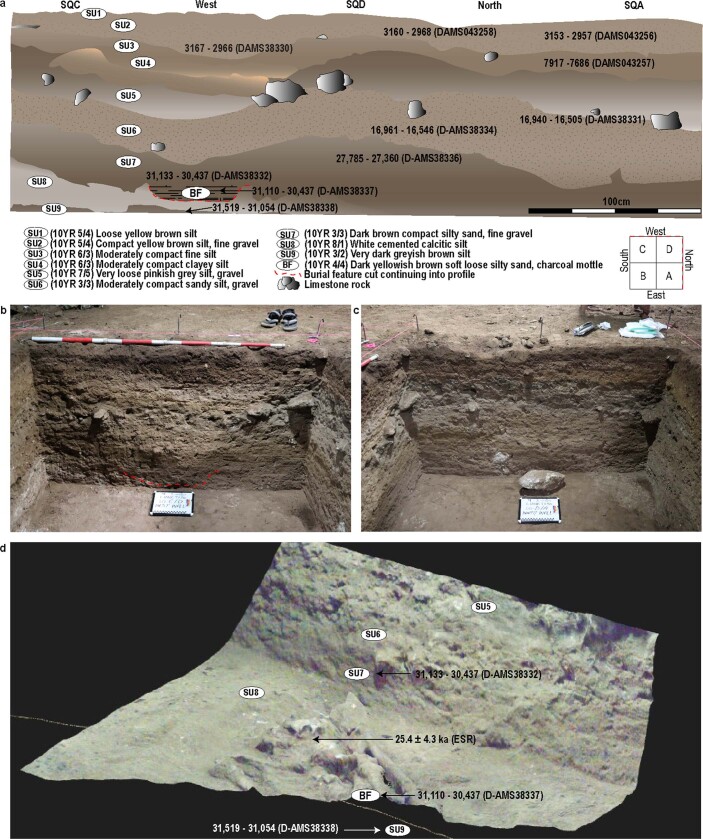
Liang Tebo stratigraphic profile. **a**, Nine major stratigraphic units (SU) with mostly horizontal bedding and strongly to moderately interpretable boundaries shown across west and north profiles. Sediments were assessed and described using Munsell colour^57^; compaction (cemented, weakly cemented, moderate, loose to very loose); and sorting grades of well (<0.5 mm), moderate (~1 mm), poor (>1.5 mm), and very poor (>2 mm) particles. Calibrated radiocarbon ages and laboratory codes are presented within respective SUs represented in stratigraphic profile. **b**, Squares C and D, west wall profile showing burial feature cut that continues into the profile. **c**, Squares D and A, north wall profile. **d**, 3D laser scan depicting thorax of TB1 skeleton, with stratigraphic units above the feature (SU5,6,7) depicted on the western wall, and a boundary line of the underlying strata SU9.

The Sangkulirang–Mangkalihat Peninsula of East Kalimantan (Indonesian Borneo) is host to an extensive limestone karst landscape (around 4,200 km^2^) that, during the Late Pleistocene, was located close to the extreme easternmost edge of the Eurasian continental landmass, Sunda (Fig. 1a). This rugged karst terrain harbours numerous caves and rock shelters that abound with archaeological evidence of prehistoric human occupation, including figurative rock art dating to at least 40 thousand years ago^4^. However, a considerable gap in Pleistocene archaeological records, particularly of human skeletal remains^5–10^, exists in the region. Liang Tebo—a large three-chambered limestone cave (around 160 m^3^) with preserved rock art in the uppermost chamber—is situated approximately 2.5 km from, and 165 m above, the Marang River (Fig. 1b and Extended Data Fig. 1). In 2020, after a geophysical survey, a 2 m by 2 m trench was excavated in the central floor area of the largest chamber of this cave. This area was excavated to a depth of 1.5 m without reaching bedrock, revealing nine major stratigraphical units (SU) and a burial feature comprising a fully articulated single adult inhumation (designated TB1), first exposed at 0.87 m depth in squares C and D (Extended Data Fig. 2).

## Burial feature

**Fig. 2:**
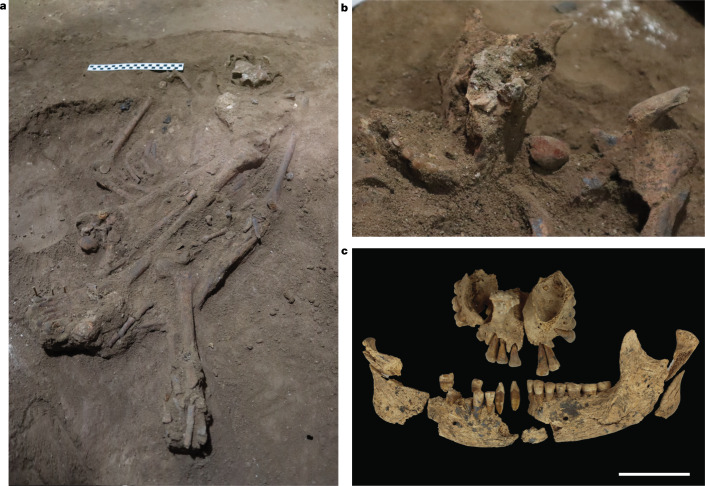
Liang Tebo burial feature. **a**, A single adult inhumation (TB1). The skull is to the right of the scale bar, as shown by the exposure of the supraorbital ridge. A flexed burial position with the right knee brought to the chest and a complete right foot, and the left knee flexed below the pelvis, with the tibia and fibula underneath the femur. **b**, In situ nodule of red ochre (a natural earth pigment) next to the mandible. **c**, Maxilla and mandible. Scale bar, 5 cm.

**Extended Data Fig. 3:**
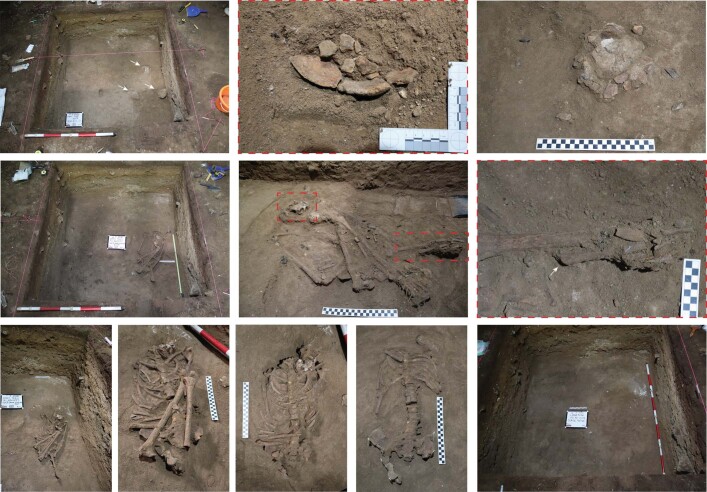
TB1 burial feature. Figure viewed from left to right, upper to lower. The TB1 burial feature was carefully excavated in 32 episodic stages (R1 to R32). Upper left, large limestone burial markers positioned above the skull and arms post-burial. Centre, skull (left red bounding box) and left femur (right red bounding box) under which amputated tibia and fibula lie. Middle right, TB1 amputated tibia shown in situ (white arrow). Lower right, burial feature post-removal demonstrating distinct grave cut margins.

The Liang Tebo burial feature exhibited a strongly defined stratigraphic boundary and distinctive infilling sediment (grave fill), showing that the grave cuts into and modifies SU8. The bottom of the ovate-shaped grave cut terminated in SU8 and did not continue into the underlying SU9 (Extended Data Fig. 2). A portion of the western margins of the burial cut was clearly visible when partially cross-sectioned by the western excavation wall (Extended Data Fig. 2). Limestone rocks were positioned above the head and each arm of the individual, immediately atop the grave infill (Extended Data Fig. 3). These apparent burial markers, coupled with strong feature boundaries, which were unique to all other associated horizontal strata (Extended Data Figs. 2 and 3), confirm that the burial was a ‘manufactured’ stratum and a deliberate human grave^11–13^. TB1 was interred lying on their back in an almost north-to-south alignment (310° N), with the left and right legs flexed—the right with the knee at the chest, and the left knee flexed below the pelvis (underneath the femur), with the left hand inferior and the right superior, to the pelvic girdle (Fig. 2a). Minimal movement of fragile bone elements suggests rapid sedimentation and decomposition within a confined space^12,13^. Cultural materials recovered from the burial include flaked chert artefacts and a 22 mm by 17 mm nodule of red ochre (a natural earth pigment), which was recovered near the mandible (Fig. 2b).

The TB1 burial feature and skeleton was removed in 32 episodic stages (R1–R32), each accompanied by laser scanning and photography (Extended Data Fig. 3). TB1 is well preserved (Supplementary Information): the reassembled skeleton reveals 75% bone presence, with all teeth present and intact (Fig. 2c and Extended Data Fig. 6), and is therefore considered relatively complete in terms of representation of the skeletal elements and the condition of bone. The individual is classified as an anatomically modern human (*Homo sapiens*) based on a range of morphological considerations (Supplementary Information). The combination of epiphyseal fusion, pubic symphysis, and auricular surface stages, as well as analyses using dental formation techniques, indicate that TB1 was a young adult, approximately 19–20 years of age at the time of death (Supplementary Information). The cranium and pelvis show intermediate sex traits and therefore the sex is indeterminate (Supplementary Information). The TB1 individual is typical in stature when compared with other prehistoric male individuals with morphological and morphometric affinity to pre-Last Glacial Maximum skeletons from Asia, and is more than one standard deviation (*σ*) taller than the mean for most female individuals (Supplementary Table 1).

## Dating

Immediately above the sediment of the distinct grave cut in SU7, a charcoal sample returned an accelerator mass spectrometry (AMS) radiocarbon (^14^C) age of 31,133 to 30,437 calibrated radiocarbon years before present (cal. bp) with a 95.4% probability (D-AMS38332), providing a stratigraphic minimum date for the inhumation of TB1 (Supplementary Table 2). In addition, a charcoal sample within the burial feature, collected from the pelvic girdle, returned an estimate of 31,110 to 30,437 cal. bp (D-AMS38337). Charcoal recovered from SU9, the stratum underlying the burial feature, provides a stratigraphic maximum date, with an estimate of 31,519 to 31,054 cal. bp (D-AMS38338). The SU9 sample was situated immediately underneath the burial cut, although within a completely distinct stratum that clearly underlies both the burial feature and the equally distinct sediments of SU8. Thus, associated radiocarbon dating of the charcoal samples indicates an age estimate for the TB1 burial feature of between 31,519 and 30,437 cal. bp, with a mean of 30,978 cal. bp. Bayesian chronology suggests that the boundary between SU7, which caps the burial, and the burial feature itself, is 30,853 ± 770 cal. bp; and the boundary between the burial and the underlying SU8 is 31,135 ± 864 cal. bp (Extended Data Fig. 10 and Supplementary Table 3). Furthermore, radiocarbon dating from overlying stratigraphic units confirms subsequent human occupation at the site transitioning the Last Glacial Maximum and the Holocene towards the surface (Supplementary Table 2), with the depth measurement of each sample showing strong and significant correlation with the mean calibrated age (*r* = 0.990, *r*^ 2^ = 0.981, *F* = 253.942, *p* = 0.001). The positive age–depth relationship of these samples (completely lacking inversion) supports an argument for minimal deposit reworking and diminishes the possibility of introduced charcoals entering lower units, including burial-fill sediments.

In addition to radiocarbon dating of the charcoal, a combined uranium-series and electron spin resonance dating technique was undertaken on a sample of TB1’s left mandibular molar (M_3_) and this analysis returned an age estimate of 25.4 ± 4.3 thousand years old (1*σ*), which is within the error of the ^14^C burial-context age. Both the uranium-series analysis in isolation and radiocarbon dating of the skeletal remains were unsuccessful owing to insufficient amounts of uranium and collagen in the sample, respectively. Incorporating the electron spin resonance age into the Bayesian model gives a modelled date of 31,201 to 30,714 years ago (2*σ* or 95.4% probability) for the burial (Extended Data Fig. 10). In summary, we infer a secure Late Pleistocene age of between 31,000 and 30,000 years for TB1, making this, to our knowledge, the oldest intentional primary burial of a modern human currently known from Island Southeast Asia.

## Evidence of surgical amputation

**Fig. 3:**
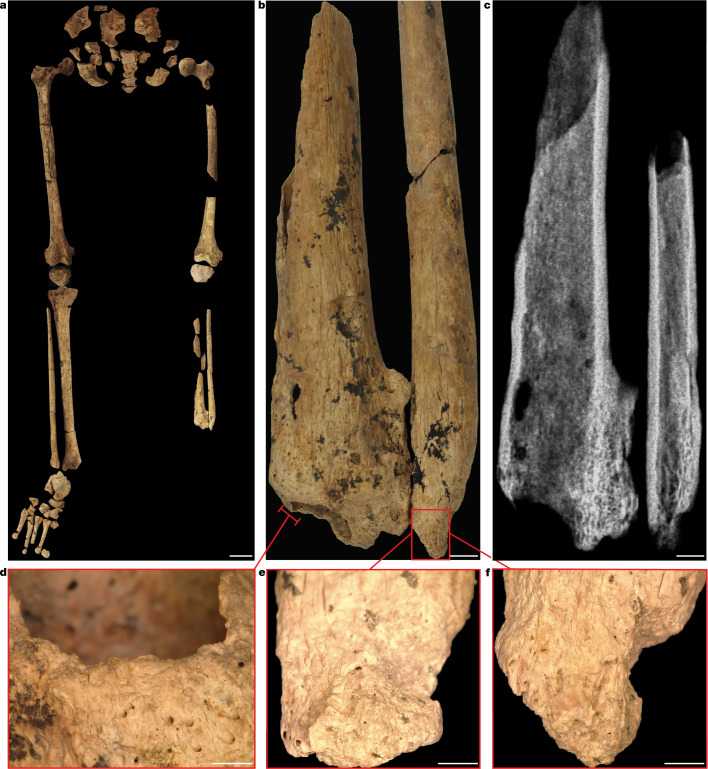
Surgically amputated site of the left tibia and fibula. **a**, TB1 left and right legs with pelvic girdle, demonstrating the complete absence of the distal third of the left lower leg. **b**, Left tibia and fibula showing the amputation surface, atrophy and necrosis. The bone surface is more porous because lysis occurred to remove the dead bone (necrosis). **c**, Radiograph of the left tibia and fibula. **d**–**f**, Remodelled bone covering the amputation surfaces, demonstrating healing after the amputation. **d**, Left tibia medial aspect. **e**, Left tibia medial aspect. **f**, left fibula anterior aspect. Images in **d**–**f** taken using an Olympus DSX1000 digital microscope. Scale bars, 5 cm (**a**), 5 mm (**b** and **c**) and 2 mm (**d**–**f**).

**Extended Data Fig. 4:**
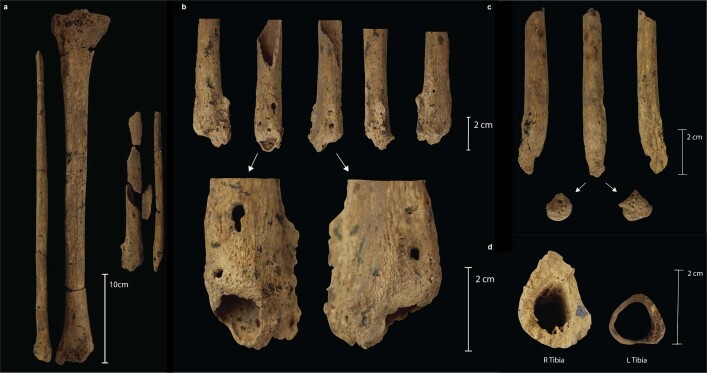
TB1 amputation. **a**, Comparison of left and right tibiae and fibulae. **b**, Left tibia showing healed amputation surfaces of amputation site. **c**, Left fibula showing healed amputation surfaces of amputation site. **d**, Cortex thickness of the left and right tibia are presented for comparison demonstrating atrophy of the lower left limb in association with the amputation.

**Extended Data Fig. 5:**
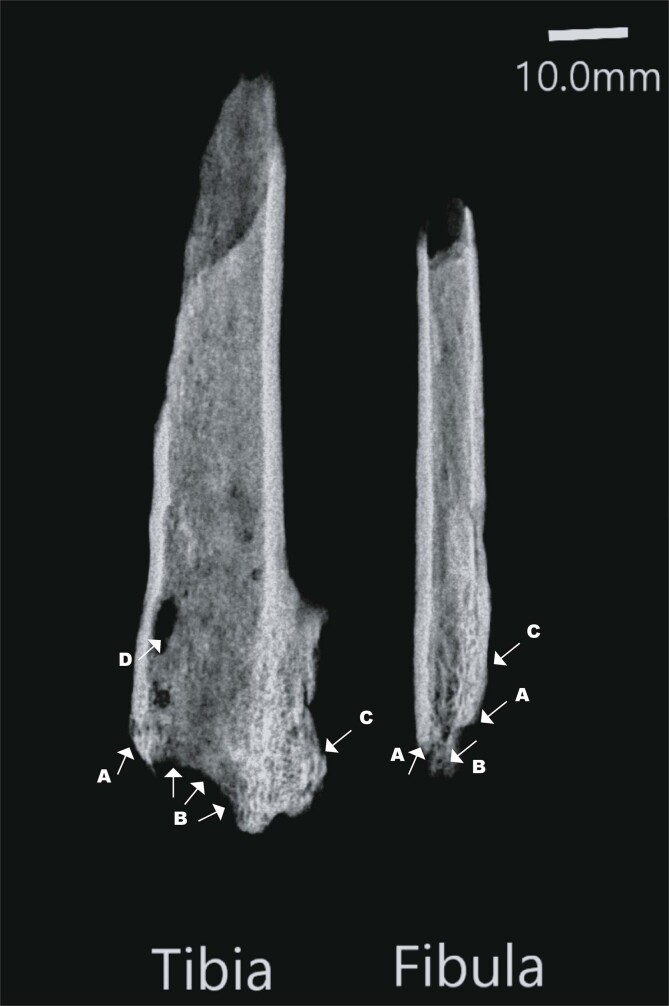
Radiograph of TB1 amputated distal left limb. White arrows demonstrate (A) Clear cutting margins indicating use of a sharp instrument. (B) Resorption of the bone due to lack of proper vascularisation of the bone tissue. (C) Heterogenic ossification of the interosseus membrane. (D) Post-mortem insect damage. Note: fibula is placed postero-anteriorly.

**Extended Data Fig. 6:**
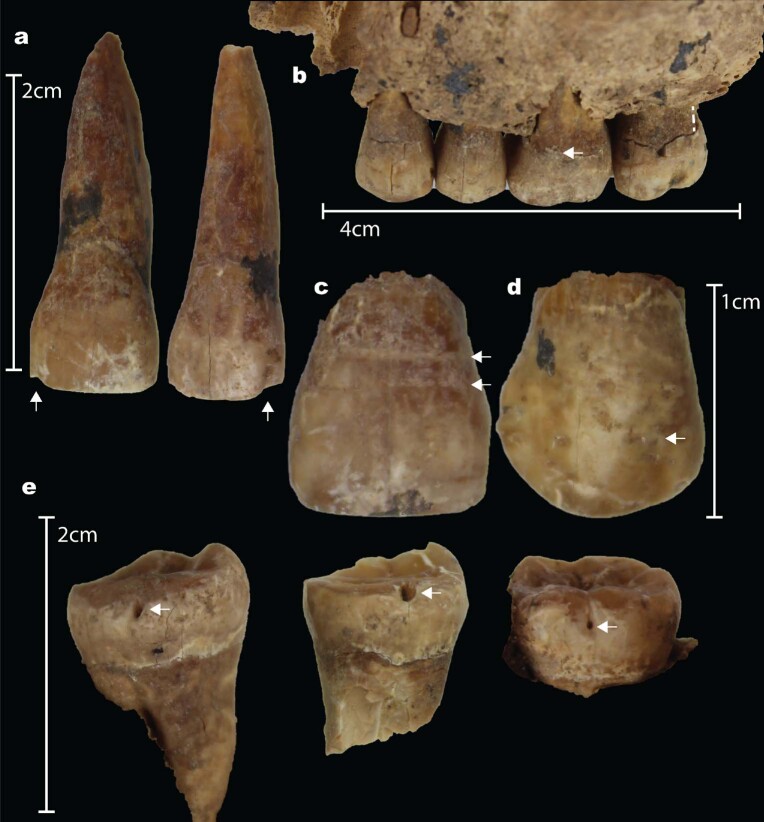
Dental pathology of TB1. **a**, notches (arrows) on the left maxillary central and lateral incisors. **b**, dental calculus (arrows) and periodontal disease (dashed line) of the left maxillary 1st premolar to 2nd molar. **c**, Linear Enamel Hypoplasia of the right maxillary central incisor. Two defects are observable. **d**, Pitted hypoplasia (arrow) of the right maxillary canine. **e**, Carious lesions (arrows) on the buccal surfaces of right mandibular 3rd molar, right mandibular 2nd molar and left mandibular 3rd molar (left to right).

Careful excavation of the burial feature containing TB1 revealed the complete absence of the left foot (Fig. 3 and Extended Data Figs. 3 and 4). Recovered left tibia and fibula shaft fragments, found flexed underneath the left femur, presented unusual distal bony growth (Fig. 3 and Extended Data Figs. 4 and 5). The opposite leg was articulated, with all right foot bones (*n* = 26) recovered within the grave (Fig. 3a). Remodelled bone covers the amputation surfaces identified on the left distal tibia and fibula shaft fragments, demonstrating healing (Fig. 3b–f, Extended Data Figs. 4 and 5 and Supplementary Information). This indicates that the distal third of TB1’s lower leg was removed through deliberate surgical amputation at the position of the distal tibia and fibula shafts. The trauma pattern observed  is not consistent with clinical descriptions of non-surgical amputation, except in cases of modern trauma in which a large metal blade or a mechanical process has been involved^14–17^. Non-surgical amputations, commonly as a result of accidents, do not cause clean oblique sectioning and are not clinically recorded to sever the lower limb of both the tibia and fibula, as is the case for TB1. Blunt-force trauma from an accident or an animal attack typically causes comminuted and crushing fractures^18^, features that are absent from the clearly simple and oblique amputation margin of TB1. Amputation as punishment is considered unlikely, particularly given the careful treatment of the individual in life after the amputation and in burial, which is not consistent with someone considered deviant^19^. Completely remodelled lamellar bone has enclosed the inferior margin of the fibula (Fig. 3e,f), indicating that TB1 died a minimum of 6–9 years after the initial trauma—confirming that this was not a fatal pathology^20–22^. There is no evidence of infection in the left limb, the most common complication of an open wound without antimicrobial treatment. The lack of infection further rules out the probability of animal attack, such as a crocodile bite, because an attack has a very high probability of complications from infection owing to microorganisms from the animal’s teeth entering the wound^23^. The partial consolidation of the bone between the left tibia and fibula and complete closure of the distal end of the left fibula (Fig. 3b,e,f and Extended Data Figs. 4 and 5) are consistent with late-stage amputation changes^14^. The small size of the left tibia and fibula compared with the right suggests a childhood injury, as the bones did not continue growing (Fig. 3a). The severe bone thinning of the left tibia and fibula is also suggestive of the heavily restricted use of the left leg resulting in musculoskeletal disuse atrophy^22^ (Extended Data Fig. 4). Some thinning of the cortical margins of the right tibia suggests that TB1 was rarely ambulatory owing to the incapacitating nature of the injury to the lower left leg (Extended Data Fig. 4).

## Discussion

The surgical amputation of TB1’s left lower leg some 31,000 years ago has important implications for our understanding of the evolution of human medico-socio-cultural practices. Evidence of surgery in the time before written records is scarce. Until now, the earliest primary evidence of advanced medical knowledge, including amputation, was restricted to Holocene cases^1,24,25^—earlier reports of deliberate amputation of limbs among Neanderthals are now considered inconclusive, although these cases remain examples of medical care intervention^26^ (for example, the malformed forelimb of Shanidar 1, is equally likely to have occurred from progressive loss of limb to disease or by accident). Furthermore, it has long been a commonly held view among western scholars that healthcare systems and medical procedures of historically known foraging societies are, and were, rudimentary. It is recognized that traditional healing practices typically involve extensive knowledge of plant-based medicinal remedies^27^. Surgical intervention and treatment of people with illness or injury, however, are thought to have been poorly developed among small-scale foraging communities, and were generally limited to procedures such as suturing lacerations, dentistry, cranial trepanation and various body modification practices such as tooth avulsion, scarification, and genital mutilation (for example, circumcision), each of which no doubt requiring considerable expertise. The prevailing assumption has been that more complex surgeries were beyond the abilities of foraging societies past and present. The surgical removal of body parts, specifically, is thought to have been confined mostly to phalangeal (finger segment) amputation for punishment or symbolic purposes (that is, as a cultural marker or mourning rite)^28^. Concerning the history of amputation surgery per se, historical accounts vary from ancient Roman sources to advances in surgical procedures developed during the past few centuries^1^. Review of the latter^1,27^ provides details of modern clinical procedures of amputation, exemplifying the level of anatomical understanding, hygiene, surgical skill, and required apparatus for success (the latter being synonymous with survival of the person with illness or injury). In western societies, successful surgical amputation only became a medical norm within the past 100 years^1^. Before modern clinical developments, including antibiotics, it was widely thought that most people undergoing amputation surgery would have died, either at the time of amputation from blood loss and shock or from subsequent infection—scenarios that leave no skeletal markers of advanced healing.

**Extended Data Fig. 7:**
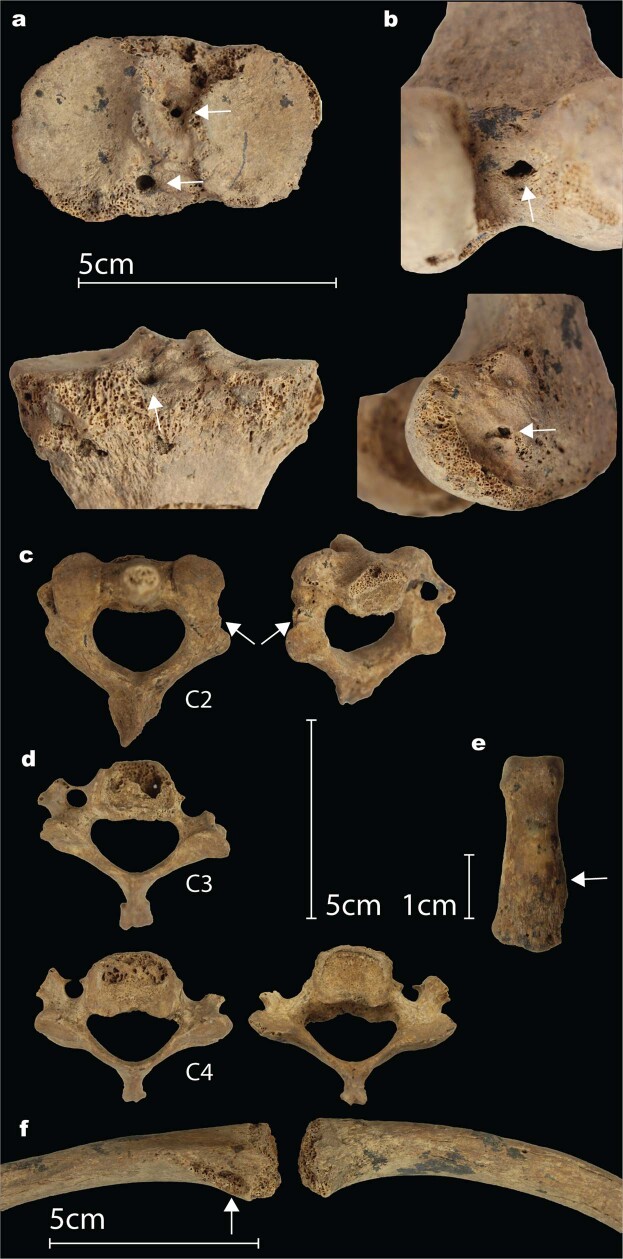
Other trauma. **a**, Osteolysis of the right proximal tibia (superior view). **b**, Osteolysis of the right distal femur (posteroinferior view). **c**, Healed fracture of the C2 right pars interarticularis (superior and inferior views). **d**, DJD of the C3 (superior view) and C4 (superior and inferior view) due to malalignment of C2 fracture. **e**, Potential fracture or new bone deposit on the left 4^th^ interproximal digital phalanx (dorsal view). **f**, Unilateral muscle activity of the clavicles (inferior view). The right clavicle is associated with a deep costoclavicular sulcus indicating muscle strain associated with repetitive rotary motion of the right shoulder.

**Extended Data Fig. 8:**
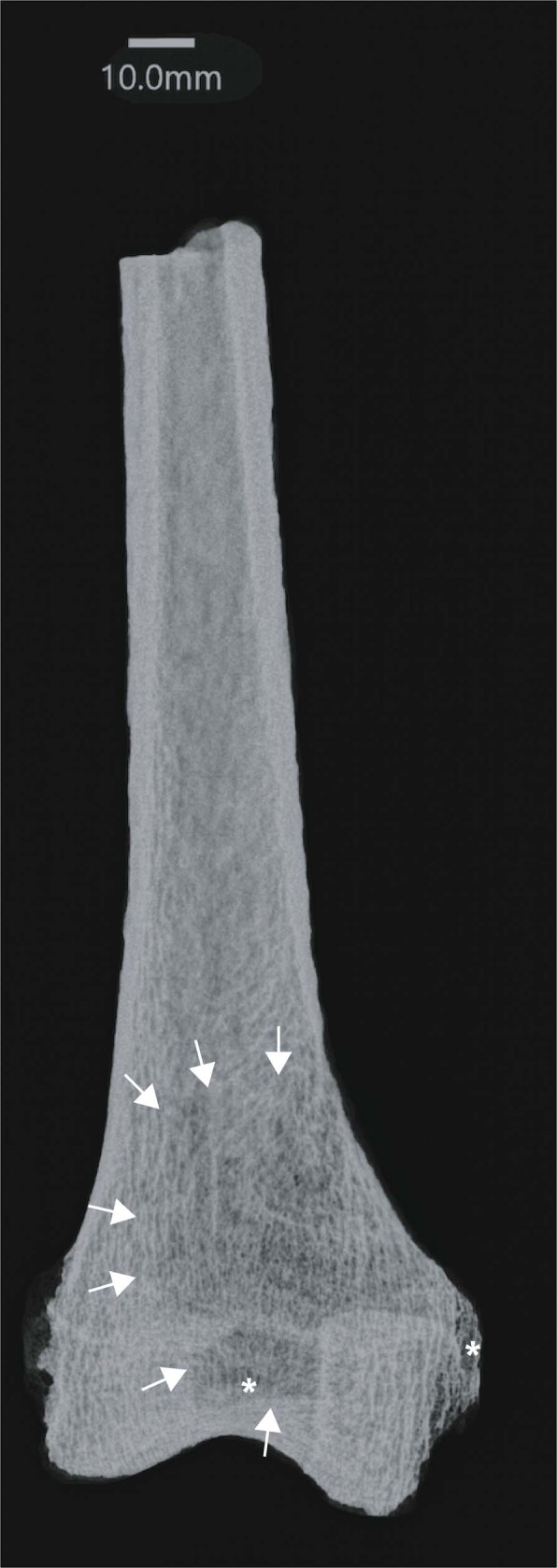
Radiograph of osteomyelitis of the distal right femur. Note: the distinct radiolucent region that extends to the margins of the lysis that is macroscopically visible on the external bone indicated by asterisk (*). The margins of the lesion (white arrows) are irregular consistent with an active and fast spreading infection. There is too much post-mortem damage to the underlying cancellous bone of the proximal tibia and the same lysis cannot be clearly observed as with the femur.

**Extended Data Fig. 9:**
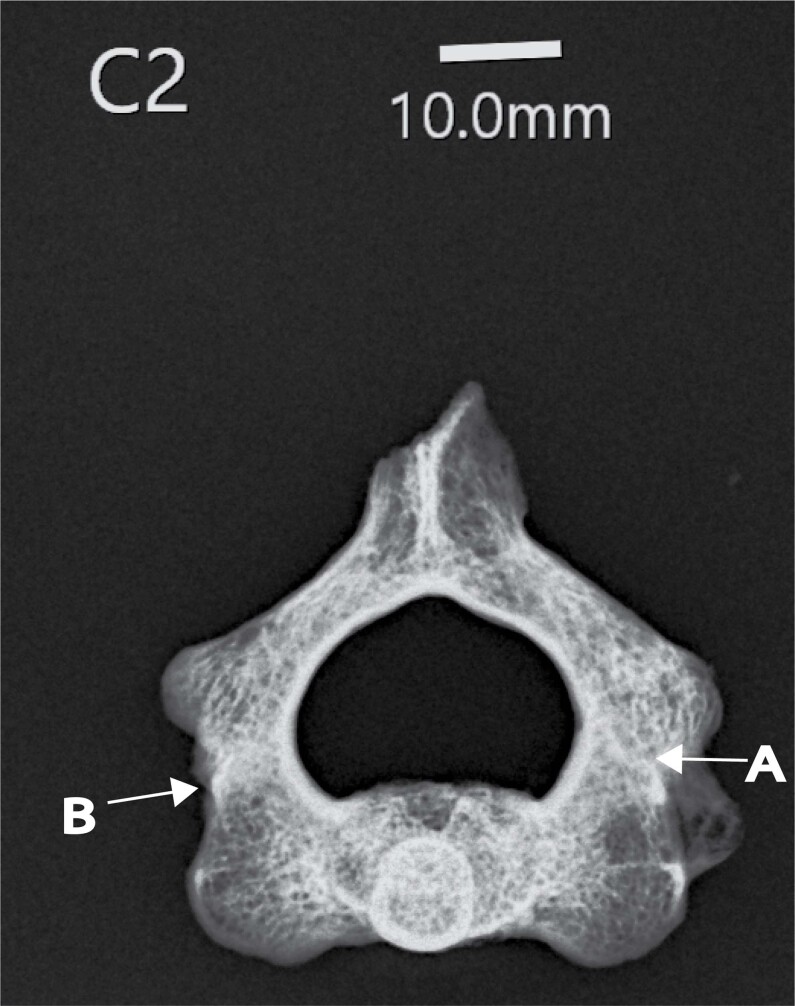
Antemortem fracture of the C2 denoted by white arrows. (A) is not macroscopically visible and more consistent with an infracture. (B) is macroscopically visible and of great consequence to deformity of the original bone shape than (A).

With regard to TB1, we infer that the Late Pleistocene ‘surgeon(s)’ who amputated this individual’s lower left leg must have possessed detailed knowledge of limb anatomy and muscular and vascular systems to prevent fatal blood loss and infection. They must also have understood the necessity to remove the limb for survival^29^. Finally, during surgery, the surrounding tissue including veins, vessels and nerves, were exposed and negotiated in such a way that allowed this individual to not only survive but also continue living with altered mobility. Intensive post-operative nursing and care would have been vital, such as temperature regulation, regular feeding, bathing, and movement to prevent bed sores while the individual was immobile^29^. The wound would have been regularly cleaned, dressed, and disinfected, perhaps using locally available botanical resources with medicinal properties to prevent infection and provide anaesthetics for pain relief ^30,31^. Although it is not possible to determine whether infection occurred after the surgery, this individual evidently did not suffer from an infection severe enough to leave permanent skeletal markers and/or cause death. Furthermore, it is inferred that life without a lower limb (combined with other traumas; Extended Data Figs. 7–9 and Supplementary Information) in a rugged and mountainous karst terrain presented a series of practical challenges—several of which can be assumed to have been overcome by a high degree of community care^32,33^.

In summary, the discovery of this exceptionally old evidence of deliberate amputation demonstrates the advanced level of medical expertise developed by early modern human foragers in a Late Pleistocene tropical rainforest environment^34^ on the eastern margins of Sunda. We infer that the comprehensive knowledge of human anatomy, physiology, and surgical procedures evident in TB1’s community is likely to have been developed by trial and error over a long period of time and transmitted inter generationally through oral traditions of learning. Notably, it remains unknown whether this ‘operation’ was a rare and isolated event in the Pleistocene history of this region, or if this particular foraging society had achieved an unusually high degree of proficiency in this area. Risk of death from trauma and disease has always been with us, and complex medical acts, such as limb amputation, could well have been more commonplace in the pre-agricultural past of our species than is broadly assumed at present. Our understanding of this aspect of *H.* *sapiens* prehistory, however, may be affected by poor preservation of pathological bone, as well as by preconceptions about the ‘primitive’ nature of earlier medico-socio-cultural practices, especially among non-sedentary foraging populations in tropical Asia. On the other hand, we cannot exclude the possibility that human colonization of the ancient rainforests of Borneo both prompted and facilitated early advances in medical technology that were unique to this region. For example, rapid rates of wound infection in the tropics may have stimulated the development of new pharmaceuticals (for instance, antiseptics) that harnessed the medicinal properties of Borneo’s rich plant biodiversity and endemic flora^30,31^.

## Methods

### Ground-penetrating radar

The geophysical survey using ground-penetrating radar (GPR) and electrical resistivity tomography (ERT) was conducted in the chambers of Liang Tebo (Extended Data Fig. 1). GPR data were collected using a Malå X3M with a 500-Mhz antenna using a time window of 62 ns with 1,024 samples, a trace interval of 2 cm and 4 stacks. GPR data were processed using ReflexW software with a suite of filters, including ‘move start time’, ‘dewow’, ‘energy decay’, ‘bandpass butterworth’, ‘background remove’ and ‘time cut’. ERT data were collected using a ZZ Flash Res-64 using an electrode spacing of 0.5 m, collected in Wenner and dipole–dipole arrays with *k* values of 20 and a dipole–dipole *l* value of 5. ERT data were acquired with 120 V, an on-time of 1.2 s and an off-time of 0.2 s. Data were output using ZZ RData Check software, inverted in Res2D using the robust scheme and displayed with a colour scale constructed using the Jenks Breaks feature with ArcGIS.

### Excavation

Sedimentary features within the deposit and all other sediment changes were excavated separately following the stratigraphical boundaries. Homogenous sediments, when encountered, were excavated in arbitrary excavation units, measuring between 1 cm and 5 cm in thickness. Materials and sedimentary features were recorded with three-dimensional plotting and laser scanning, using a Leica MS60 Robotic Total Station. All artefacts larger than around 19 mm in maximum dimension were plotted in three dimensions and all stratigraphic features were laser-scanned. All sediments were sieved using 1.5-mm screens, while feature sediments (including those surrounding the burial) were sieved using a soft nylon 0.5-mm screen. Whether recovered in situ or from sieved residues, all artefacts can be precisely associated with both a stratigraphical unit and an excavation unit. Cultural materials recovered throughout include stone artefacts, ochre, shell, faunal remains and macrobotanical remains, with a total lack of ceramic and metal finds. Human remains and all other delicate artefacts were excavated using handheld softwood tools to prevent damage, with other sediments removed using a fine leaf trowel. First encountered at 0.87 m depth in the western squares, the TB1 burial feature had a strongly defined stratigraphical boundary with distinctive infill sediment: revealing the grave cuts into SU8. The latter unit was marked by a very different colour and texture—a weakly cemented white (10YR 8/1) calcitic silt (Extended Data Fig. 2)—making grave cut boundaries particularly distinctive (Extended Data Fig. 3). The thin western margins of the burial cut were partially cross-sectioned by the western excavation wall and served to define these stratigraphical relationships in profile (Extended Data Fig. 2). Feature boundaries of the burial were unique to surrounding and overlying strata, constituting a ‘manufactured’ stratum^13^ that modified SU8. These observations rule out that the body was placed into natural crevices or deposited through natural processes^12,35^ and, instead, support an interpretation of a deliberately excavated grave cut into SU8. Placement of large stratigraphically analogous limestone rocks as burial markers (Extended Data Fig. 3) further distinguished the upper surface of the grave and supports the case of deliberate burial. A red ochre (earth pigment) nodule adjacent to TB1’s mandible on the left clavicle (Fig. 2b) is likely to be a mortuary good placed near the mouth. Anatomical integrity and articulation of unstable joints, the first to decompose, support a primary and relatively undisturbed burial (Fig. 2a).

### Dating

Throughout the nine stratigraphical units (Extended Data Fig. 2), a total of 10 in situ radiocarbon dating samples (charcoal plotted three-dimensionally during excavation) were dated using AMS ^14^C dating at the Direct AMS laboratory, in Seattle, USA (Supplementary Table 3). Dates were calibrated using OxCal (v.4.4), with the Northern Hemisphere atmospheric curve (IntCal20)^36^. Samples were pretreated using acid–base–acid protocols. Samples were incubated in 6 M HCl at 65 °C for 12 min and rinsed with deionized water, incubated again in 6 M HCl at 65 °C for 12 min and rinsed 3 times with deionized water, incubated in 0.09 M KOH at 65 °C for 12 min and rinsed in deionized water, and then rinsed with 0.05 M HCl. This base step with subsequent rinses was repeated twice more. Finally, the pretreatment was finished with 2 additional 0.05 M HCl rinses. Samples D-AMS 038331 and D-AMS 038334 received additional base step(s), for a total of 4 and 5 steps, respectively. Sample D-AMS 038338 showed signs of breakdown in base and thus received a less-aggressive acid–base–acid base step, using 0.09 M KOH at room temperature for 12 min followed by a deionized water rinse and 0.05 M HCl rinse, and treatment with 0.09 M KOH at 65 °C for 12 min and a deionized water rinse and, finally, washed using three 0.05 M HCl rinses. Carbon δ^13^C stable isotope values are not available for these samples.

**Extended Data Fig. 10:**
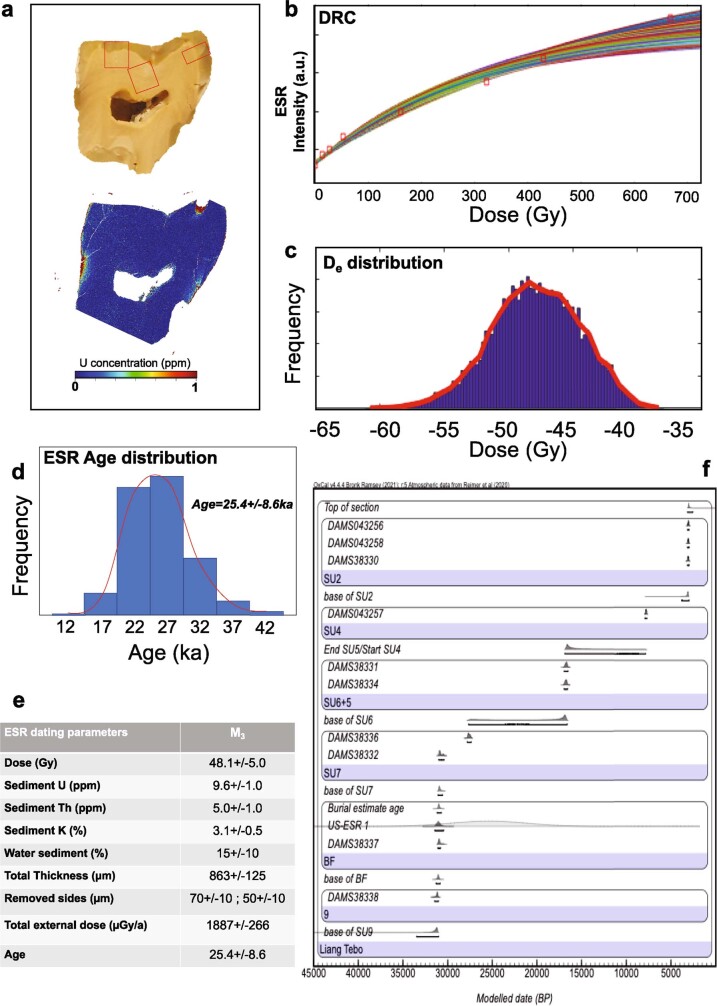
Direct US-ESR dating of mandibular left third molar (M_3_) and dating sequence. **a**, Tooth section and polished Uranium-series analyses (red rectangles indicate location of laser rasters), with Trace elemental map: distribution of uranium in the M_3_ dental tissues. **b**, Dose response curve (DRC) obtained using McDoseE 2.0 program^60^ with an iteration of 100,000. **c**, Dose equivalent D_e_ frequency distribution; **d**, Age distribution frequency for the third molar (M_3_). **e**, Table summarising parameters and data used for modelling the ESR age (2-sigma). **f**, Results of the Bayesian model age sequence.

Coupled uranium-series and electron spin resonance (US-ESR) dating was done on a left mandibular molar (M_3_) at the GARG facility of the Southern Cross University. The tooth was first cut in half using a rotating diamond saw with a blade of 300 µm, before being polished to 5-µm smoothness. The sample was then analysed for uranium-series isotopes and concentration in both dentine and enamel using a laser ablation NWR ESI 213 laser coupled with a MC–ICPMS Neptune XT (Thermo Fisher) to calculate the internal dose rate. An enamel fragment was then measured on a Freiberg MS5000 ESR X-band spectrometer and irradiated with the Freiberg X-ray irradiation chamber. ESR intensities were extracted from the merged spectra obtained on the angular variation measurements^37^ (Extended Data Fig. 10), after correcting for the baseline, subtraction of isotropic signals and assessment of the NOCORS contribution using the published protocol^38,39^ (Extended Data Fig. 10). Dose–response curves were obtained using the MCDOSE 2.0 software^40^ (Extended Data Fig. 10). All age calculations were carried out with the DATA program^41^.

Bayesian modelling was performed on all age estimates using OxCal (v.4.4)^36^. The analysis incorporated the probability distributions of individual dates and constraints imposed by stratigraphical relationships. The model was structured using phases and boundaries in a contiguous pattern, with each stratigraphical unit representing a separate phase (Extended Data Fig. 10 and Supplementary Table 3). The aim of the modelling was to estimate the age of the boundaries between each stratigraphical unit on the basis of the dating results obtained for that unit. No attempt was made to remove identified outliers. This is because we do not know the underlying ‘true’ age depth model and we are using different dating methods, so it is difficult to specify the criterion to identify true outliers. Instead of this approach, we have explicitly specified minimum and maximum ages where appropriate to do so, in keeping with the nature of the dating methods and the quality of the results. We believe that this is a better method compared with an outlier analysis in this context, as it avoids unnecessary bias (that is, in the choice of criterion) and represents a more-conservative approach.

The age estimates are coeval and the uncertainties are relatively small. As such, the identified boundary ages are not sensitive to removal of individual dates or to changes in, for example, the model calculation resolution. None of the changes we made to the model set-up produced appreciable differences in the age model results. The age of the burial layer was conservatively estimated as the boundary between the base of this layer and the base of SU7, incorporating all of the constraints described above and the resulting age estimates (Extended Data Fig. 10 and Supplementary Table 3).

### Osteology

Bone preservation was assessed both in terms of completeness (how much of the skeleton was present) and taphonomy (post-depositional processes that have affected the bones). Skeletal and dental completeness and post-depositional processes, including colour change, root damage, animal scavenging marks, sun and water exposure, post-mortem breakage and surface erosion, were each assessed^42,43^.

The TB1 individual was morphologically an adult, therefore adult age-at-death estimation techniques were applied. Pubic symphysis and auricular surface degeneration stage methods were compared with standards^44,45^. Different fusion timings of the various epiphyses enable a narrow age estimate of late teenage years to early adulthood. Epiphyses (growth plates) that do not fuse until early adulthood, such as the medial end of the clavicle, were assessed following previously published studies^46^. Dental eruption, wear and formation methods supplemented these age-estimation protocols^47–51^.

Regression equations were used to estimate the stature from the maximum length of the long bones. The right femur and tibia were considered the most valuable bones for stature estimation because of their relationships in contributing to stature and preservation. Australo-Melanesian populations rather than East or southeast Asian populations are likely to provide better estimates for pre-Neolithic individuals from southeast Asia. The ‘American Black’ stature estimate standards were used^52,53^ because of the similar proportions to the contribution of maximum tibia lengths, with 10 mm adjustments to the maximum tibial lengths^52^. Estimates for comparative pre-Neolithic hunter-gatherers in southeast Asia have traditionally been estimated from modern Asian populations in the United States, even if they pre-date migration of groups with morphological affinity to modern East Asian populations to the region. Therefore, these stature estimates are provided for comparison to other pre-Neolithic modern humans.

A full skeletal assessment of abnormal bone changes was completed. Lesions (any pathological bone loss, growth or deformity) were recorded following revised standard protocols^54–56^. Bone lesion location, aspects affected, percentage of bone affected by lesion and bone type affected (cortical, trabecular and/or medullary canal) were recorded to assess the spatial distribution of lesions. The level of healing, margin definition, presence of necrotic bone (sequestrum), presence of shape changes to the bone, focality (focal, multifocal or diffuse), laterality, symmetricity and lesion size were recorded to reconstruct the progression and pattern of disease for differential diagnosis. Lesions were compared against clinical and palaeopathological literature to determine possible candidates for disease origin (aetiology of the disease). Trauma analysis (for example, fractures) followed previously published protocols^56^ to describe the mechanism of injury, force, type and time of trauma, and the degree and complications to healing.

### Reporting summary

Further information on research design is available in the Nature Research Reporting Summary linked to this article.

## Online content

Any methods, additional references, Nature Research reporting summaries, source data, extended data, supplementary information, acknowledgements, peer review information; details of author contributions and competing interests; and statements of data and code availability are available at 10.1038/s41586-022-05160-8.

## Supplementary information

Supplementary Information Additional notes on the Liang Tebo skeleton’s preservation, age-at-death and sex (p.1), analysis of the amputation site and dental pathology (p.2) and other trauma (p.3). Additional notes on the US–ESR dating analyses (p.5–8). Supplementary Tables 1–4 (p.9–12) include the stature estimates, linear enamel hypoplasia details, radiocarbon chronology and the Bayesian model and code, respectively.

Reporting Summary

## Data Availability

All data generated or analysed during this study are included in the published Article (and its Supplementary Information).

## Code availability

All code used in this study is provided in Supplementary Table 3.

## References

[1] Roberts, C. A. in *The Archaeology of Medicine* (ed. Arnott, R.) Vol. 1046, 1–20 (Archaeopress, 2002).

[2] Richards MP (2002). A brief review of the archaeological evidence for Palaeolithic and Neolithic subsistence. Euro. J. Clin. Nutrition.

[3] Buquet-Marcon, C., Philippe, C. & Anaick, S. The oldest amputation on a Neolithic human skeleton in France. *Nat. Prec.*10.1038/npre.2007.1278.1 (2007).

[4] Aubert M (2018). Palaeolithic cave art in Borneo. Nature.

[5] Brumm A (2021). Skeletal remains of a Pleistocene modern human *(Homo sapiens)* from Sulawesi. PLoS ONE.

[6] O’Connell J (2018). When did *Homo sapiens* first reach Southeast Asia and Sahul?. Proc. Natl Acad. Sci. USA.

[7] Oxenham, M. & Buckley, H. *The Routledge Handbook of Bioarchaeology in Southeast Asia and the Pacific Islands* (Routledge, 2016).

[8] Samper-Carro SC (2021). Burial practices in the early mid-Holocene of the Wallacean Islands: a sub-adult burial from Gua Makpan, Alor Island, Indonesia. Quat. Int..

[9] Curnoe D (2016). Deep skull from Niah Cave and the Pleistocene peopling of Southeast Asia. Front. Ecol. Evol..

[10] Westaway KE (2017). An early modern human presence in Sumatra 73,000–63,000 years ago. Nature.

[11] Pettitt, P. *The Palaeolithic Origins of Human Burial* (Routledge, 2011).

[12] Gargett RH (1999). Middle Palaeolithic burial is not a dead issue: the view from Qafzeh, Saint-Césaire, Kebara, Amud, and Dederiyeh. J. Hum. Evol..

[13] Martinón-Torres M (2021). Earliest known human burial in Africa. Nature.

[14] Barber CG (1929). Immediate and eventual features of healing in amputated bones. Ann. Surg..

[15] Donnally III C (2018). Orthopedic injuries associated with jet-skis (personal watercrafts): a review of 127 inpatients. Orthop. Traumatol. Surg. Res..

[16] Pennoyer GP (1930). Traumatic amputation of the thigh, complicated by both tetanus and gas gangrene with recovery. J. Am. Med. Assoc..

[17] Sherk VD (2008). BMD and bone geometry in transtibial and transfemoral amputees. J. Bone Miner. Res..

[18] Aydin K, Cokluk C (2007). A fracture of unilateral pars interarticularis of the axis: a case report. Turk. Neurosurg..

[19] Mavroforou A (2014). Punitive limb amputation. Clin. Orthop. Relat. Res..

[20] Lovell NC (1997). Trauma analysis in paleopathology. Am. J. Phys. Anthropol..

[21] Wendeberg B (1961). Mineral metabolism of fractures of the tibia in man studied with external counting of Sr85. Acta Orthop. Scand..

[22] Sievänen H (2010). Immobilization and bone structure in humans. Arch. Biochem. Biophys..

[23] Wamisho BL (2009). Ward round-crocodile bites in Malawi: microbiology and surgical management. Malawi Med. J..

[24] Juengst SL, Chavez SJ (2015). Three trepanned skulls from the Copacabana Peninsula in the Titicaca Basin, Bolivia (800 bc–ad 1000). Int. J. Paleopathol..

[25] Zhou Y (2020). Early evidence of trepanation along the Yellow River Basin in Neolithic China. Archeol. Anthropol. Sci..

[26] Spikins P (2019). Living to fight another day: the ecological and evolutionary significance of Neanderthal healthcare. Quat. Sci. Rev..

[27] Ackerknecht EH (1947). The role of medical history in medical education. Bull. Hist. Med..

[28] Burns, K. R. *Forensic Anthropology Training Manual* (Routledge, 2015).

[29] Wen APY (2020). Successful ankle replantation in two cases with different presentations. Arch. Plast. Surg..

[30] Az-Zahra FR (2021). Traditional knowledge of the Dayak Tribe (Borneo) in the use of medicinal plants. Biodiversitas.

[31] Gibbons, S. & Teo, S. P. (eds) *Medicinal Plants of Borneo* (CRC, 2021).

[32] Oxenham MF (2009). Paralysis and severe disability requiring intensive care in Neolithic Asia. Anthropol. Sci..

[33] Tilley L, Oxenham MF (2011). Survival against the odds: modeling the social implications of care provision to seriously disabled individuals. Int. J. Paleopathol..

[34] Wurster CM (2019). Savanna in equatorial Borneo during the late Pleistocene. Sci. Rep..

[35] Pettitt, P. *The Palaeolithic Origins of Human Burial* (Routledge, 2011).

[36] Reimer PJ (2020). The IntCal20 Northern Hemisphere radiocarbon age calibration curve (0–55 cal kBP). Radiocarbon.

[37] Joannes-Boyau R, Grün R (2011). A comprehensive model for CO_2_^−^ radicals in fossil tooth enamel: implications for ESR dating. Quat. Geochron..

[38] Grün R, Aubert M, Joannes-Boyau R, Moncel MH (2008). High resolution analysis of uranium and thorium concentrations as well as U-series isotope distributions in a Neanderthal tooth from Payre using laser ablation ICP-MS. Geochim. Cosmochim. Acta..

[39] Joannes-Boyau R (2013). Detailed protocol for an accurate non-destructive direct dating of tooth enamel fragment using electron spin resonance. Geochronometria.

[40] Joannes-Boyau R, Duval M, Bodin T (2018). MCDoseE 2.0. A new Markov chain Monte Carlo program for ESR dose response curve fitting and dose evaluation. Quat. Geochron..

[41] Grun R (2009). The DATA program for the calculation of ESR age estimates on tooth enamel. Quat. Geochron..

[42] Buikstra, J. E. & Ubelaker, D. H. *Standards for Data Collection from Human Skeletal Remains* (Archaeological Survey Research Series 44, 1994).

[43] McKinley, J. in *Guidelines to the Standards for Recording Human Remains* (eds Brickley, M. & McKinley, J.) Ch. 5, 14–17 (BABAO, Institute of Field Archaeologists, 2004).

[44] Brooks, S. & Suchey, J. M. Skeletal age determination based on the os pubis: a comparison of the Acsádi-Nemeskéri and Suchey-Brooks methods. *Hum. Evol.* **5**, 227–238 (1990).

[45] Lovejoy CO (1985). Chronological metamorphosis of the auricular surface of the Ilium: a new method for the determination of adult skeletal age at death. Am. J. Phys. Anthropol..

[46] Schaefer, M., Black, S. M. & Scheuer, L. *Juvenile Osteology: A Laboratory and Field Manual* (Elsevier, Academic, 2009).

[47] Ubelaker, D. H. *Human Skeletal Remains: Excavation, Analysis, Interpretation* (Taraxacum, 1989).

[48] Moorrees CF, Fanning EA, Hunt EE (1963). Age variation of formation stages for ten permanent teeth. J. Dent. Res..

[49] Scott EC (1979). Dental wear scoring technique. Am. J. Phys. Anthropol..

[50] Reid DJ, Dean MC (2000). Brief communication: the timing of linear hypoplasias on human anterior teeth. Am. J. Phys. Anthropol..

[51] Reid DJ, Dean MC (2006). Variation in modern human enamel formation times. J. Hum. Evol..

[52] Trotter M, Gleser GC (1952). Estimation of stature from long bones of American whites and Negroes. Am. J. Phys. Anthropol..

[53] Jantz LM, Jantz RL (1999). Secular change in long bone length and proportion in the United States, 1800–1970. Am. J. Phys. Anthropol..

[54] Littleton, J. & Kinaston, R. in *Forensic Approaches to Death, Disaster and Abuse* (ed. Oxenham, M.) 155–176 (Australian Academic Press, 2008).

[55] Buckley, H. R. *Health and Disease in the Prehistoric Pacific Islands* (British Archaeological Reports International Series 2792, 2016).

[56] Ortner, D. J. *Identification of Pathological Conditions in Human Skeletal Remains* (Academic, 2003).

[57] Munsell Color Co. Inc. *Munsell Soil Color Charts* (1992).

