# Supplementary material for: Surgical amputation of a limb 31,000 years ago in Borneo
Source: Nature. 2022 Sep 7;609(7927):547–51. doi: 10.1038/s41586-022-05160-8 (PMC9477728; doi:10.1038/s41586-022-05160-8)
Supplement: Supplementary file 1 — Additional notes on the Liang Tebo skeleton’s preservation, age-at-death and sex (p.1), analysis of the amputation site and dental pathology (p.2) and other trauma (p.3). Additional notes on the US–ESR dating analyses (p.5–8). Supplementary Tables 1–4 (p.9–12) include the stature estimates, linear enamel hypoplasia details, radiocarbon chronology and the Bayesian model and code, respectively. [file 41586_2022_5160_MOESM1_ESM.pdf]

nature portfolio

https://doi.org/10.1038/s41586-022-05160-8

Supplementary information

Surgical amputation of a limb 31,000 years ago in Borneo

In the format provided by the
authors and unedited

Surgical amputation of a limb 31,000 years ago in Borneo

Tim Ryan Maloney[1,2*], India Ella Dilkes-Hall[3*], Melandri Vlok[4*], Adhi Agus Oktaviana[5,6*], Pindi Setiawan[7*], Andika Arief Drajat Priyatno[8*], Marlon Ririmasse[9], I. Made Geria[9], Muslimin A.R. Effendy[8], Budi Istiawan[8], Falentinus Triwijaya Atmoko[8], Shinatria Adhityatama[5,6], Ian Moffat[10], Renaud Joannes-Boyau[11,12], Adam Brumm[2], Maxime Aubert[1,2,11*]

[1] Griffith Centre for Social and Cultural Research, Griffith University, Gold Coast, Queensland, Australia.

[2] Australian Research Centre for Human Evolution, Griffith University, Nathan, Queensland, Australia.

[3] Archaeology, School of Social Sciences, University of Western Australia, Crawley, Western Australia, Australia.

[4] Sydney South East Asian Centre, University of Sydney, New South Wales, Australia.

[5] BRIN, OR Arkeologi, Bahasa dan Sastra, Pusat Riset Arkeometri, Jakarta, Indonesia.

[6] School of Humanities, Languages and Social Science, Griffith University, Gold Coast, Queensland, Australia.

[7] Art and Design Faculty, Bandung Institute of Technology, Bandung, Indonesia.

[8] Balai Pelestarian Cagar Budaya Kalimantan Timur, Samarinda, Indonesia.

[9] BRIN, OR Arkeologi, Bahasa dan Sastra, Pusat Riset Lingkungan, Maritim, dan Budaya Berkelanjutan, Jakarta, Indonesia.

[10] Archaeology, College of Humanities, Arts and Social Sciences, Flinders University, Bedford Park, South Australia, Australia.

[11] Geoarchaeology and Archaeometry Research Group (GARG), Southern Cross University, Lismore, New South Wales, Australia.

[12] Palaeo-Research Institute, University of Johannesburg, Gauteng Province, South Africa.

[*]These authors contributed equally to this work.

The SI contains additional notes on the Liang Tebo skeleton’s preservation, age-at-death, sex (p.2), analysis of amputation site, dental pathology (p.3), and other trauma (p.4). Additional notes are provided on Uranium-Series and Electron Spin Resonance dating (p.5-8). Supplementary data tables 1, 2, 3 and 4 are also presented (p.9 – 12); summarising stature estimates, Linear Enamel Hypoplasia details, radiocarbon chronology, as well as the Bayesian model and code.

Liang Tebo articulated single adult skeleton (TB1)

Preservation

TB1's bones were reddish brown in appearance due to the post-depositional burial environment. Surfaces varied from McKinley[66] Grade 1-2 minimal (axial skeleton, scapula and pelvis, hand, and feet bones) to Grade 3-4 (large limb bones), with widespread erosion of the bone surfaces. Canine puncture marks were present on multiple skeletal elements including the skull, vertebrae, and long bones. Rodent gnawing and beetle chewing/boring was common on muscle attachments of the long bones. The burial environment preserved trabecular bone, the most fragile bone which is commonly destroyed in post-depositional environments. Some cracking and flaking of the forearm bones is present, possibly due to drying of the bone during the process of excavation.

Age-at-death

The preserved right pubic symphysis and left and right auricular surfaces presented with no degeneration (stage 1) indicating the individual was under 24 years of age. All epiphyses were fused with the exception of partially fused ischial rami and S1-2 bodies, and unfused medial clavicular and manubrial 1st costal notch epiphyses. Third molars were all erupted, roots complete and in occlusion, with the exception of the left maxillary M_3 which had erupted less than 1 mm short of occlusion. The combination of these age-at-death methods provided a narrow estimation between 19–20 years of age.

Sex

In comparison to the stature of other pre-LGM skeletons, TB1's is typical for males and more than 1 standard deviation (SD) taller than females (Extended Data Table 1). A narrow greater sciatic notch and the lack of a preauricular sulcus was suggestive of a male[67]. While the epiphyses of the iliac crest and ischial tuberosity demonstrated fusion common at the termination of puberty, the pelvis was morphologically typical of pre-pubescence, and abnormally small in shape. Under-development of the pelvis and therefore development of secondary sex characteristics was possibly disrupted. As the female pelvis develops female features from a 'male-like' pre-pubescent form, the presence of male traits in an under-developed pelvis cannot be considered as skeletal evidence that the individual was male. The cranium displayed more feminine or intermediate traits and presented with gracile supraorbital, mandibular and occipital bone features. A Walrath score of -0.8 indicates overall more feminine than masculine cranial features. In contrast to the pelvis, the male cranium develops more robust muscle attachments during puberty from a 'female-like' pre-pubescent form due

to testosterone production[66]. Therefore, as is the case with sex estimation of the pelvis, it is not possible to determine whether these features are secondary sex characteristics or default skull traits due to impaired pubertal development. Estimating sex along a binary, when in reality sex is expressed along a spectrum of biological and morphological traits influenced by genetics, environment and hormonal development, is a well-recognised limitation in bioarchaeology[68.] Therefore, sex is 'indeterminate' for this individual.

Macroscopic and radiographic analysis of amputation site

TB1 exhibited antemortem oblique trauma to the distal third shafts of the left tibia and fibula (Fig. 3). The trauma is in the remodelling phase of healing indicating this is an old pathology. The remodelling encloses the inferior margin of the bone indicating non-union with the distal tibia. Radiographs demonstrate clean margins consistent with surgical amputation. Compression necrosis at the amputation surface, reveals restricted blood flow and disrupted healing, indicates the Tebo individual applied pressure to the left lower limb and likely used it as a stump[69, 70] (Fig. 3b,c; Extended Data Fig. 4 and 5).

Additionally, a bony extension between the fibula and tibia known as heterogenic ossification is present, which may be a result of the amputation or as a result of soft or hard tissue trauma to the area during and after the event that required acute cure. The maximum thickness of the cortex of the left tibia was 1.89 mm compared to 5.69 mm at the same region (distal third tibia) for the right tibia, indicating that the use of the limb was severely restricted for a long period of time leading to atrophy[69-75]. Similar severe atrophy was observed in a man with quadriparesis from Neolithic Vietnam, with restricted use of his limbs since adolescence[71,72]. The amputation is not consistent with clinical descriptions of traumatic amputation, instead with surgical amputation, except in cases of trauma where a blade has been involved[75-77]. Although there are signs of weight bearing use of the left limb, TB1 is unlikely to have been an independently mobile adult amputee.

Dental pathology

Linear enamel hypoplasia was identified in most anterior teeth (Extended Data Fig. 6; Extended Data Table 4), with macroscopic evidence for five stress events across multiple teeth, occurring approximately at 1.9-2 years, 2.3-2.4 years, 2.9-3.1 years, 3.2-3.6 years, and 4.4-4.6 years. Physiological stress at these ages are not uncommon, corresponding with significant changes in sociocultural development, growth, diet, and maturation of the immune system[78]. White opacities were also observed across multiple anterior and posterior teeth, indicating episodes

of non-specific stress affecting enamel formation during the entire period of the development of permanent dentition (Extended Data Fig. 6d). Mild to medium periodontal disease was present along the alveolar bone (gum line) of posterior teeth (Extended Data Fig. 6b). There was no associated ante mortem tooth loss or other evidence of the mandible or maxillary bony palate disease. Mild dental calculus was observed in posterior teeth (Extended Data Fig. 6b). Carious lesions were observed in three mandibular molars (Extended Data Fig. 6e). Comparatively low levels of carious lesion development may suggest a relatively low cariogenic diet (hard fibrous instead of sugary/starchy foods)[78,79]. 'Notching' of the maxillary left central and lateral incisors were also observed (Extended Data Fig 5a), often associated with the use of 'teeth as tools', particularly in the processing of fibres[80,81].

Other trauma

Two circular osteolytic lesions with rounded margins of the proximal right tibia (Extended Data Fig. 7a), and two similar osteolytic lesions of the adjoining region of the right distal femur, measuring between 3.4 x 3.4 mm to 5.9 x 3.8 mm, are present (Extended Data Fig. 7b). These lesions are reminiscent of cloacae[82], pus draining holes that form due to osteomyelitis (infection of bone). As the cloacae face the knee joint, it is likely the joint was infected which is a common occurrence in long bone osteomyelitis[83]. The right patella has no abnormal bone changes. The most common cause of osteomyelitis is *Staphylococcus aureus*, and chronic bone osteomyelitis generally forms over months or years[83]. The infection has caused large osteolysis in the epiphyses of the proximal tibia and distal femur (Extended Data Fig. 8). There is no evidence of bone death (sequestrum) nor associated involucrum, a shaft of new bone that forms in response to bone destruction and necrosis commonly found alongside cloacae, indicating the bone infection has not progressed to late stages. There is no remodelling of the internal osteolysis observed via radiograph indicating the infection was active at time of death.

A remodelled fracture of the C2 right pars interarticularis was also observed (Extended Data Fig. 7c,d and 9). This fracture is an extremely rare type of spondylolisthesis (vertebral ring fracture) also known as a 'hangman's fracture' as these can be caused by the knot of a noose during executions. Unilateral expression of this fracture is rare. Clinically, they are reported from car accidents where the direct mechanism of injury is the acceleration of the body forward with a sudden stop causing hyperextension at the upper neck joint, or due to compression with a slight rotation of the neck[84-85]. A force of between 840 to 1220 N is needed to cause such a fracture[85]. A 60 kg person would meet this threshold of force from a 1.4–2 m free fall with a

sudden stop on the head and neck. Hyperflexion or hyperextension of the neck with falls can occur from height, and when tossed by a wild animal such as a water buffalo[86,87].

This remodelled fracture healed partially misaligned. This misalignment laterally displaced the inferior articular facet which affected the direction of facing articular surfaces between the C2 and C3. This displacement caused subsequent degenerative joint disorder (DJD) of the superior vertebral body of the C3 and the superior and inferior vertebral body of the C4. DJD presented as marginal osteophytes, porosity and new bone of the joint surfaces. It is not possible to determine if the cause of injury leading to the C2 fracture and the amputation was from a single event. However, both are injuries which occurred well before death. Minor trauma was observed on the right clavicle with a unilateral development of a subclavian sulcus (Extended Data Fig. 7f), a nonmetric trait associated with strain of the right shoulder in a rotary motion[72].

Uranium-Series and Electron Spin Resonance dating

The sample used for the coupled U-series and ESR dating analyses is a mandibular left third molar (M_3) which was well preserved including partial dental roots. The tooth was sectioned to expose the different dental tissues using a diamond blade-rotating saw with a thickness of 300 µm. The tooth was then pre-screened by LA-ICPMS quadrupole to assess the suitability of uranium-series and electron spin resonance coupled dating technique (enamel concentration <5ppm)[88-90], as well as an increase understanding of the diagenetic and diffusion processes within the sample.

A fragment of enamel was separated using a hand-held diamond saw following established protocol[60] and cleaned of any remaining dentine. Fragment was stripped of the outer ≈60 microns ±10 on each side using a polishing diamond blade. Fragment was mounted into a parafilm mould to record the angular dependency in the ESR response[90,91]. Fragment was then measured at room temperature on a Freiberg MS5000 ESR X-band spectrometer at a 0.1mT modulation amplitude, 10 scans, 2mW power, 100G sweep, and 100KHz modulation frequency for ESR dating. Irradiation was performed with the Freiberg X-ray irradiation chamber, which contains a Varian VF50 X-ray gun at a voltage of 40KV and 0.5m. A current on the fragment exposed to X-rays without shielding (apart from a 200 µm Al foil layer). Each fragment was irradiated, following exponentially increasing irradiation times (50s, 101s, 200s, 600s, 1203s, 1600s, 2500s). For each irradiation step, the energy output of the X-ray gun was recorded at the beginning and end and averaged, which allows to correct for the dose rate received by the sample (average dose rates of ~0.267 Gy/s). At the start and after each irradiation step the

fragment was measured over 180° in X, Y and Z-configurations with a 10° step (e.g., Extended Data Fig. 10). ESR intensities were extracted from T1-B2 peak-to-peak amplitudes on the merged ESR signal. Isotropic and baseline corrections were applied uniformly across the measured spectra[92-98]. The amount of NOCORs was estimated to be negligible using the protocol described by[60]. The ESR dose response curves were obtained by using merged ESR intensities and associated standard deviations from the repeated measurements over one orientation only.

Fitting procedures were carried out with the MCDOSE 2.0 software using a Markov Chain Monte Carlo (MCMC) approach based on the Metropolis-Hastings algorithm[92]. A D_E value of 48.1+/-5 Gy was obtained by fitting a single saturating exponential (SSE) at the appropriate maximum irradiation dose (D_{max}) following recommendations[95] with iteration of 10,000 counts, n burn of 25,000 and an average acceptance rate of 30.15%.

Water content in the enamel and the dentine was assumed at 3% ±1 and 5% ±3 respectively. The external dose rate values for water content and elemental content in the sediment were obtained from drying out the sediment in oven and measuring weight difference. A large error was used to account for important seasonal variation in water content.

Cosmic dose rate was estimated using burial depth and geographic location according to the DATA program[91] with a sediment density of 2.95g/cm^3. External dose rate was calculated using sediments recovered around the tooth at the time of excavation. Dried sediment was crushed before being digested in a 1:3 Nitric/Hydrochloric acid solution overnight, prior to analysing with an ICPMS quadrupole. Uranium, Thorium and Potassium concentrations were then used to extrapolate the external dose contribution assuming equilibrium of the decay chain and an assumed ^{230}Th/^{232}Th-ratio of 0.8+/-0.8. Sediment content for U, Th and K of 9.56ppm (+/-1), 5.02 ppm (+/-1) and 3.1% (+/-.5) respectively was used to calculate the external contribution to the total dose rate assuming a 30 cm homogenous sphere around the sample. US-ESR ages were modelled using both the DATA program and the US-ESR program[90], and updated dose rate conversion factors[97-98].

The tooth was initially sampled for Uranium content using LA-ICPMS quadrupole to assess the suitability of the fossil for dating before being measured by MC-ICPMS. Measurements were obtained by laser ablation, using an NW213 ESI laser coupled to a 7700 Agilent Quadrupole and a MC-ICPMS Neptune XT at Southern Cross University, GARG facility. The entire tooth was imaged for trace elements using the quadrupole setting with spot size of

40microns and a translation speed of 80microns and dwell time of 0.5s. For elemental maps obtained by LA-ICP-MS, ablation lines were extracted in the form of a .csv files generated by the MassHunter Workstation software (Agilent). Each file was then imported into the interactive R Shiny application “shinyImaging” (http://labs.icahn.mssm.edu/lautenberglab/), which transforms each isotope into a separate file containing the counts per second (CPS) values of one element and is organised as a matrix (number of ablation lines multiplied by the number of ablation spots per ablation line). For each element, gas blank CPS (median value in pixels per ablation spot) collected during the first 10s of each analysis (gas blank) were used as background and subtracted from the rasterstack. Baseline and drifts were corrected using NIST 610 and 612 glass standard. Background around the teeth (signal arising from the encasing resin) was converted to white coloration (no intensity) to increase clarity of the figures by isolating the dental tissue from its surroundings. Colour scales were applied using the linear blue-red Lookup Table.

Additionally, large areas were sampled using the LA-MC-ICPMS setting with 700microns rasters using a 110 um spots size at a 5micron per second translation speed and a fluence of ~4.88J cm^2 (Extended Data Fig. 10). Concentration and isotopic ratio for the enamel were measured serval parts of the enamel including on the fragment used for ESR measurements. Dentine values were averaged for the entire tooth. Baseline and drifts were corrected using NIST 610 and 612 glass standard, while two corals as well as a fossil Rhinoceros tooth[93] were used to correct $^{234}U/^{238}U$ and $^{230}Th/^{238}U$ ratios and assess the accuracy of measurements. Uranium elemental map reveal that the dental tissues were virtually free of uranium. Both instrumental measurements of uranium and thorium concentrations across the entire sample reached only a few ppb to tens of ppb at a maximum. It remains unclear why no diffusion occurred in the sample, perhaps the unusual yellowish coating around the tooth acted as a barrier prohibiting water and moisture to enter the sample. The absence of a measurable level of uranium and thorium in both dentine and enamel hindered any meaningful calculation of uranium-series age for the sample. Additionally, isotopic ratios $^{234}U/^{238}U$ and $^{230}Th/^{238}U$ were below detection limits and could not be produced. Consequently, the internal dose rate component of the sample was considered null and only the external dose and cosmic dose component contributed to the total annual dose for ESR age calculation. The direct dating calculation on the human mandibular left third molar propose an age for the deposition between 21.1 ka and 29.7 ka (1-sigma). Obviously with such an insignificant amount of uranium in the sample both early and linear uptake results are identical. Cosmic dose is estimated to contribute

to ~12% of the total external dose. The burial while placed inside the rock shelter is in proximity of an open shaft. The cosmic dose rate had to be extrapolated from pictures and is likely to be overestimated. Dating of the remains assuming a complete shielding of cosmic rays from the cave roofs would only place the remains closer to the radiocarbon dating, with an estimate at 27.8ka+/-4.1 (1-sigma). Since no environmental gamma dose measurements were conducted in situ, the assumption of a 30 cm sphere around the sample and the extrapolation to the external dose rate and cosmic dose rate from the sediment content corresponds to the leading and non-negligible source of error.

Supplementary Data Table 1. Stature estimates from maximum femur lengths of Pre-Neolithic and Neolithic males in East and Southeast Asia. The TB1 individual was estimated to be between approximately 168.9 cm to 175.8 cm tall (χ =172.3 cm), and 162.9 to 170.5 cm (χ = 166.7 cm) with US Black and US Asian standards respectively. In comparison to the stature of other prehistoric individuals with Australo-Melanesian ancestry, the Tebo individual is a typical stature for males and more than 1 SD taller than the mean for females.

Site	Location	Age k	χ cm.	SD	Reference
Tebo	Indonesia	31	166.7	3.8	This study
Tam Hang	Laos	15.7	166.24 *	3.8	61
Huiyaotian	South China	9-7	167.6	2.3	62
Liyupo	South China	9-7	163.3	5.2	63
Zengpiyan	South China	9-7.5	164.7	3.2	63
Pha Phen	Laos	7	175.27 *	3.8	61
Con Co Ngua	Vietnam	7-6.2	165.15-176.18 *	-	61
Man Bac	Vietnam	3.5-4	163.2	6.6	64

*Calculated using raw max length estimates. US Asian standards applied.

Supplementary Data Table 2. Liang Tebo radiocarbon chronology. Charcoal samples calibrated using OxCal v. 4.4, with the Northern Hemisphere Atmospheric curve [IntCal20][65], at Direct AMS laboratory.

Code	SQ/XU	SU	Sample	Mass (mg)	Radiocarbon age	δ13C	95.4% cal BP
DAMS043256	A/XU3#1	SU2	Charcoal	15.8	2,902±27	69.68±0.23	3,153 - 3,090 (17.9%)<br>3,083 - 2,957 (77.6%)
DAMS043258	D/XU4#1	SU2	Charcoal	40.4	2,920±24	69.52±0.21	3,160 - 2,994 (92.1%)<br>2,980 - 2,968 (3.4%)
DAMS38330	A/XU4 #2	SU2	Charcoal	17.9	2,926±28	69.47±0.24	3,167 - 2,966
DAMS043257	A/XU5#1	SU3	Charcoal	64.1	6,951±32	42.09±0.17	7,917 - 7,902 (2.8%)<br>7,855 - 7,686 (92.7%)
DAMS38331	A/XU11#1	SU6	Charcoal	41.6	13,775±49	18±0.11	16,940 - 16,505
DAMS38334	D/XU12#1	SU6	Charcoal	93.2	13,797±49	17.95±0.11	16,961 - 16,546
DAMS38336	D/XU18#1	SU7	Charcoal	26.1	23,406±104	5.43±0.07	27,785 - 27,360
DAMS38332	A/XU20#1	SU7	Charcoal	27.6	26,633±113	3.62±0.051	31,133 - 30,437
DAMS38337	D/XU19BF1	19BF	Charcoal	23.9	26,591±110	3.65±0.05	31,110 - 30,437
DAMS38338	C/XU19#1	SU9	Charcoal	35.7	27,163±135	3.4±0.057	31,519 - 31,054

Supplmenttary Data Table 3. Bayesian model and code.

code	cal age	sd	SU	Modelled (BP) 95.4%		Modelled SU (BP) 95.4%	
				From	to	From	to
-	-	-	1	-	-	3145	2,973
43256	3,020	63	2	3,147	2,966	3,793	2,973
43258	3,077	83		3,155	2,973		
38330	3,081.5	85.5		3,161	2,937		
43257	7,770.5	84.5	4	7,917	7,685	16,862	7,836
38331	16,722.5	217.5	6	16,963	16,514	27,647	16,602
38334	16,750.5	210.5		16,955	16,549		
38336	27,572.5	212.5	7	27,786	27,361	31,090	30,585
38332	30,785	348		31,013	30,403		
38337	30,773.5	336.5	BF	31,201	30,714	31,201	30,714
US/ESR	25,400	4,300		31,452	30,463		
38338	31,286.5	232.5	9	31,299	30,845	33,472	31,029

Model code:

```
Plot()
{Sequence("Liang Tebo")
{Boundary("base of SU9");
Phase("9")
{R_Date("DAMS38338", 27163, 135);
};Boundary("base of BF");
Phase("BF")
{R_Date("DAMS38337", 26591, 110);
Date("US-ESR 1", N(calBP(25400), 4300));
Date("Burial estimate age");};
Boundary("base of SU7");
Phase("SU7")
{R_Date("DAMS38332", 26633, 113);
R_Date("DAMS38336", 23406, 104);};
Boundary("base of SU6");
Phase("SU6+5")
{R_Date("DAMS38334", 13797, 49);
R_Date("DAMS38331", 13775, 49);};
Boundary("End SU5/Start SU4");
Phase("SU4")
{R_Date("DAMS043257 ", 6951, 32);};
Boundary("base of SU2");
Phase("SU2")
{R_Date("DAMS38330 ", 2926, 28);
R_Date("DAMS043258 ", 2920, 24);
R_Date("DAMS043256 ", 2902, 27);};
Boundary("Top of section");};};
```

Supplementary Data Table 4. Linear Enamel Hypoplasia (LEH) in TB1 skeleton.

Dental Arcade Quadrant	Tooth	LEH Defects	Defect Length (mm)	Approximate age (years)*
Right Maxilla	Central Incisor	2	5.91<br>7.62	2.2-2.4[1]<br>2.9-3.4[2]
	Lateral Incisor	3	4.9<br>7.01<br>8.27	2.92<br>3.5-3.7 (min) 3.9-4.1 (max)[3]<br>4.4-4.64
	Canine	2	4.56<br>6.83	3.1-3.42<br>3.5-3.8 (min) 4.0/4.3 (max)[3]
Left Maxilla	Central Incisor	2	4.4<br>5.66	1.9-2.0[5]<br>2.2-2.4[4]
	Canine	2	3.94<br>6.12	2.3-2.4(min) 2.5-2.7 (max)[1]<br>2.8-3.0 (min) 3.1-3.4 (max)[2]
Right Mandible	Central Incisor	2	3.96<br>4.70	1.5-1.7 (min) 1.7 2.0 (max)[5]<br>2.0-2.31
	Lateral Incisor	3	3.6<br>6.47<br>6.90	1.7-1.8 (min) 1.9 -2.1 (max)[5]<br>2.6-2.8 (min) 3.0-3.2 (max)[2]<br>3.0-3.3 (min) 3.4-3.7 (max)[3]
	Canine	2	4.59<br>7.04	3.2-3.63<br>3.7-4.2 (min) 4.2-4.9 (max)[4]
Left Mandible	Lateral Incisor	2	5.83<br>6.39	2.6-2.8<br>3.0/3.3 (min) 3.4-3.7 (max)[3]
	Canine	2	3.65<br>4.95	2.4-2.71<br>3.2-3.63

*Superscript numbers indicate relation to same physiological stress event based on approximate age of the LEH defect on the tooth.

Supplementary Information References

59. Munsell Color Co. Inc. *Munsell Soil Color Charts*. New Windsor, New York. (1992).

60. Joannes-Boyau, R., Duval, M., & Bodin, T. MCDoseE 2.0. A new Markov Chain Monte Carlo program for ESR dose response curve fitting and dose evaluation. *Quat. Geochron*. **44**, 13-22 (2018).

61. Tayles, N. et al. A prehistoric flexed human burial from Pha Phen, Middle Mekong Valley, Laos: Its context in Southeast Asia. *Anthropol. Sci.* **123**, 1-12 (2015).

62. Matsumura, H. et. al. *Bio-anthropological Studies of Early Holocene Hunter-Gatherer Sites at Huiyaotian and Liyupo in Guangxi, China*. National Museum of Nature and Science, Tokyo (2017).

63. Institute of Archaeology (CASS), Guangxi Archaeological Team, Zengpiyan Museum & Guilin Archaeological Team. *Guilin Zengpiyan (The Zengpiyan Cave in Guilin)*. Cultural Relics Publishing House, 497-499 (2003).

64. Matsumura, H. et al. Morphometric affinity of the late Neolithic human remains from Man Bac, Ninh Binh Province, Vietnam: Key skeletons with which to debate the 'Two Layer' Hypothesis. *Anthropol. Sci*. **116**, 135-148 (2008).

65. Reimer, P.J. et al. The IntCal20 Northern Hemisphere Radiocarbon Age Calibration Curve (0–55 cal kBP). *Radiocarbon* **62**(4), 725-757 (2020).

66. McKinley, J. Compiling a Skeletal Inventory: Disarticulated and co-Mingled remains, in: Brickley, M., McKinley, J. (Eds.), Guidelines to the Standards for Recording Human Remains. BABAO/ Institute of Field Archaeologists, Reading (2004).

67. Lovejoy, C.O. et al. Chronological metamorphosis of the auricular surface of the Ilium: A new method for the determination of adult skeletal age at death. *Am. J. Phys. Anthropol*. **68** 15-28 (1985).

68. Brooks, S. Suchey, J.M. Skeletal age determination based on the Os Pubis: A comparison of the Acsádi-Nemeskéri and Suchey-Brooks methods. *J. Hum. Evol*. **5**, 227-238 (1990).

69. Young, D. et al. Tibial changes in experimental disuse osteoporosis in the monkey. *Calcified tissue int.* **35**, 304-308 (1983).

70. Barber, C.G. Immediate and eventual features of healing in amputated bones. *Ann. Surg.* **90**, 985 (1929).

71. Oxenham, M. et al. Paralysis and severe disability requiring intensive care in Neolithic Asia. *Anthropol. Sci*. **117**, 107-112 (2009).

72. Tilley, L. Oxenham, M.F. Survival against the odds: Modeling the Social Implications of Care Provision to Seriously Disabled Individuals. *Int. J. Paleopath.* **1,** 35-42 (2011).

73. Young, D. et al. Tibial changes in experimental disuse osteoporosis in the monkey. *Calcified Tissue Int.* **35**, 304-308 (1983).
74. Sherk, V.D. et al. BMD and bone geometry in transtibial and transfemoral amputees. *J Bone Min. Res.* **23**, 1449-1457 (2008).
75. Donnally III, C. et al. Orthopedic injuries associated with jet-skis (personal watercrafts): A review of 127 patients. *Ortho. & Trauma. Surg. Res*. **104**, 267-271 (2018).
76. Pennoyer, G.P. Traumatic amputation of the thigh, complicated by both tetanus and gas gangrene with recovery. *J. Ameri. Med. Ass.* **95**, 342-343 (1930).
77. Aydin, K., Cokluk, C.A. Fracture of unilateral pars interarticularis of the axis: A case report. *Turkish Neurosurgery* **17**, 155-157 (2007).
78. Hillson, S. Dental Anthropology. Cambridge University Press (1996).
79. Gagnon, C.M. Exploring oral paleopathology in the Central Andes: A review. International journal of paleopathology **29**, 24-34 (2020).
80. Larsen, C.S. Dental modifications and tool use in the western Great Basin. American Journal of Physical Anthropology **67**, 393-402 (1985).
81. Vlok, M., Paz, V., Crozier, R., Oxenham, M.F. A New Application of the Bioarchaeology of Care Approach: A Case Study from the Metal Period, the Philippines. International Journal of Osteoarchaeology **27**, 662-671 (2017).
82. Lew, D.P., Waldvogel, F.A. Osteomyelitis. The Lancet **364**, 369-379 (2004).
83. Lewis, M.E.. Paleopathology of Children: Identification of Pathological Conditions in the Human Skeletal Remains of Non-Adults. Academic Press, London (2017).
84. Fielding, J.W. et al. Traumatic spondylolisthesis of the axis. *Clin. Ortho. Related Res.* 47-52 (1989).
85. Teo, E.C. et al. Experimental investigation of failure load and fracture patterns of C2 (axis). *J. Biomechanics* **34**, 1005-1010 (2001).
86. Scott, R.M. et al. Domestication and large animal interactions: Skeletal trauma in northern Vietnam during the hunter-gatherer Da But Period. *PloS one* **14**, (2019).
87. Spiotta, A.M., Matoses, S.M. Neurosurgical considerations after bull goring during festivities in Spain and Latin America. *Neurosurgery* **69**, 455-461 (2011).
88. Duval, M. Evaluating the accuracy of ESR dose determination of pseudo-Early Pleistocene fossil tooth enamel samples using dose recovery tests. *Radiation Measurements* **79**, 24-32 (2015).
89. Duval, M., & Grün, R. Are published ESR dose assessments on fossil tooth enamel reliable? *Quaternary Geochronology* **31**, 19-27 (2016).

90. Joannes-Boyau, R., Grün, R. Decomposition of beta-induced ESR spectra of fossil tooth enamel. *Radiation Physics and Chemistry* **80**, 335-342 (2011).
91. Joannes-Boyau, R., Bodin, T., Grün, R. Decomposition of the angular ESR spectra of fossil tooth enamel fragments. *Radiation Measurements* **45**, 887-898 (2010).
92. Grün, R., Mahat, R., Joannes-Boyau, R. Ionization efficiencies of alanine dosimeters and tooth enamel irradiated by gamma and X-ray sources. *Radiation Measurements* **47**, 665-668 (2012).
93. Joannes-Boyau, R. Detailed protocol for an accurate non-destructive direct dating of tooth enamel fragment using Electron Spin Resonance. *Geochronometria* **40**, 322-333 (2013).
94. Joannes-Boyau, R., Grün, R., Bodin, T. Decomposition of the laboratory irradiation component of angular ESR spectra of fossil tooth enamel fragments. *Applied Radiation and Isotopes* **68**, 1798-1808 (2010).
95. Joannes-Boyau, R., Grün, R. Decomposition of UV induced ESR spectra in enamel fragments of a modern and a fossil tooth. *Ancient TL* **28**, 23-34 (2010).
96. Joannes-Boyau, R., Grün, R. Thermal behavior of oriented and non-oriented CO_2^- radicals in tooth enamel. *Radiation Measurements* **44**, 505-511 (2009).
97. Shao, Q., Bahain, J. J., Dolo, J. M., & Falguères, C. Monte Carlo approach to calculate US-ESR age and age uncertainty for tooth enamel. *Quat. Geochron.* **22**, 99-106 (2014).
98. Guérin, G., Mercier, N., & Adamiec, G. Dose rate conversion factors: update. *Ancient TL* **29**,5-8 (2011).
