# Supplementary material for: Surgical amputation of a limb 31,000 years ago in Borneo
Source: Nature. 2022 Sep 7;609(7927):547–51. doi: 10.1038/s41586-022-05160-8 (PMC9477728; doi:10.1038/s41586-022-05160-8)
Supplement: Supplementary file 2 — Reporting Summary [file 41586_2022_5160_MOESM2_ESM.pdf]

nature portfolio

Corresponding author(s): Tim Maloney

Last updated by author(s): Jul 20, 2022

Reporting Summary

Nature Portfolio wishes to improve the reproducibility of the work that we publish. This form provides structure for consistency and transparency in reporting. For further information on Nature Portfolio policies, see our Editorial Policies and the Editorial Policy Checklist.

Statistics

For all statistical analyses, confirm that the following items are present in the figure legend, table legend, main text, or Methods section.

n/a	Confirmed	
☐	☒	The exact sample size (*n*) for each experimental group/condition, given as a discrete number and unit of measurement
☐	☒	A statement on whether measurements were taken from distinct samples or whether the same sample was measured repeatedly
☐	☒	The statistical test(s) used AND whether they are one- or two-sided<br>*Only common tests should be described solely by name; describe more complex techniques in the Methods section.*
☐	☒	A description of all covariates tested
☐	☒	A description of any assumptions or corrections, such as tests of normality and adjustment for multiple comparisons
☐	☒	A full description of the statistical parameters including central tendency (e.g. means) or other basic estimates (e.g. regression coefficient) AND variation (e.g. standard deviation) or associated estimates of uncertainty (e.g. confidence intervals)
☐	☒	For null hypothesis testing, the test statistic (e.g. *F*, *t*, *r*) with confidence intervals, effect sizes, degrees of freedom and *P* value noted<br>*Give P values as exact values whenever suitable.*
☐	☒	For Bayesian analysis, information on the choice of priors and Markov chain Monte Carlo settings
☐	☒	For hierarchical and complex designs, identification of the appropriate level for tests and full reporting of outcomes
☒	☐	Estimates of effect sizes (e.g. Cohen's *d*, Pearson's *r*), indicating how they were calculated

Our web collection on statistics for biologists contains articles on many of the points above.

Software and code

Policy information about availability of computer code

Data collection	Radiocarbon dates are calibrated using OxCal version 4.4, with the Northern Hemisphere Atmospheric curve [IntCal20].<br>For ground penetrating radar, data were processed using ReflexW software with a suite of filters, including Move Start time, Dewow, Energy Decay, Bandpass Butterworth, Background Remove and Time Cut.<br>ERT data collection was undertaken using a ZZ Flash Res-64 using an electrode spacing of 0.5 m, collected in Wenner and Dipole-Dipole arrays with k values of 20 and a Dipole-Dipole l value of 5.<br>Acquisition was undertaken with 120V, an on-time of 1.2 and an off-time of 0.2 seconds.<br>Data was output using ZZ RData Check software, then inverted in Res2D using the robust scheme, and displayed with a colour scale constructed using the Jenks Breaks feature with ArcGIS. For the combined US and ESR dating, the dose response curve were obtained using MCDOSE 2.0 software.
Data analysis	The full baysian model code is presented in extended data table 3.

For manuscripts utilizing custom algorithms or software that are central to the research but not yet described in published literature, software must be made available to editors and reviewers. We strongly encourage code deposition in a community repository (e.g. GitHub). See the Nature Portfolio guidelines for submitting code & software for further information.

Data

Policy information about availability of data

All manuscripts must include a data availability statement. This statement should provide the following information, where applicable:

- Accession codes, unique identifiers, or web links for publicly available datasets
- A description of any restrictions on data availability
- For clinical datasets or third party data, please ensure that the statement adheres to our policy

All data generated or analysed during this study are included in this published article (and its supplementary information files).

Field-specific reporting

Please select the one below that is the best fit for your research. If you are not sure, read the appropriate sections before making your selection.

☐ Life sciences ☒ Behavioural & social sciences ☐ Ecological, evolutionary & environmental sciences

For a reference copy of the document with all sections, see nature.com/documents/nr-reporting-summary-flat.pdf

Life sciences study design

All studies must disclose on these points even when the disclosure is negative.

Sample size	*Describe how sample size was determined, detailing any statistical methods used to predetermine sample size OR if no sample-size calculation was performed, describe how sample sizes were chosen and provide a rationale for why these sample sizes are sufficient.*
Data exclusions	*Describe any data exclusions. If no data were excluded from the analyses, state so OR if data were excluded, describe the exclusions and the rationale behind them, indicating whether exclusion criteria were pre-established.*
Replication	*Describe the measures taken to verify the reproducibility of the experimental findings. If all attempts at replication were successful, confirm this OR if there are any findings that were not replicated or cannot be reproduced, note this and describe why.*
Randomization	*Describe how samples/organisms/participants were allocated into experimental groups. If allocation was not random, describe how covariates were controlled OR if this is not relevant to your study, explain why.*
Blinding	*Describe whether the investigators were blinded to group allocation during data collection and/or analysis. If blinding was not possible, describe why OR explain why blinding was not relevant to your study.*

Behavioural & social sciences study design

All studies must disclose on these points even when the disclosure is negative.

Study description	Archaeological excavation design: Sedimentary features within the deposit, all other sediment changes, were excavated separately following stratigraphic boundaries. Homogenous sediments, when encountered, were excavated in arbitrary excavation units (XU), measuring between 1 cm and 5 cm in thickness. Materials and sedimentary features were recorded with 3D plotting and laser scanning, using a Leica MS60 Robotic Total Station. All artefacts larger than ~19 mm in maximum dimension were plotted in 3D, and all stratigraphic features were laser-scanned. All sediments were sieved using 1.5 mm screens, while feature sediments (including those surrounding the burial) were sieved using a soft nylon 0.5 mm screen.
Research sample	Ancient adult human skeleton (sex indeterminate) recovered from archaeological excavation (2x2m) in a large limestone cave, suspected to have been occupied in the past. This is the rationale.
Sampling strategy	Excavation targeted limestone caves as they often preserve archaeological stratigraphy. GPR was used to locate the deepest area.
Data collection	Archaeological excavation with soft and metal tools (trowels/sieves) recorded with 3D laser scanning total station, photography, and detailed notes.
Timing	The excavation began on 20/02/2020 and finished on 13/03/2020.<br>Analysis of the skeletal material commenced on 14/06/2021,concluding by the 28th.
Data exclusions	No data were excluded. The deposit was excavated using hand held tools, with Sedimentary features and, all sediment changes excavated separately following stratigraphic boundaries. Homogeneous sediments, when encountered, were excavated in arbitrary excavation units (XU), measuring between 1 cm and 5 cm in thickness. Materials and sedimentary features were recorded with 3D plotting and laser scanning, using a Leica MS60 Robotic Total Station. All artefacts larger than ~19 mm in maximum dimension were plotted in 3D, and all stratigraphic features were laser-scanned. All sediments were sieved using 1.5 mm screens, while feature sediments (including those surrounding the burial) were sieved using a soft nylon 0.5 mm screen.<br>All people present during the excavation are listed in the acknowledgments, including: Stephanus Gung, Unding Reski, Petrus

	Lampung, Mardan Mardhan, Aifan Gatz, Aidil Putra, Hendrick, Satriadi, Heldi, Johansyah, Yunuss Gung, Sugianoor, Su'ud, Rendi, Hendra, Ham. Ifan, Rusdi, Ali, Leo, Aping, Djoang, and Syahdan.
Non-participation	None.
Randomization	Not relevant to this archaeological study.

Ecological, evolutionary & environmental sciences study design

All studies must disclose on these points even when the disclosure is negative.

Study description	*Briefly describe the study. For quantitative data include treatment factors and interactions, design structure (e.g. factorial, nested, hierarchical), nature and number of experimental units and replicates.*
Research sample	*Describe the research sample (e.g. a group of tagged Passer domesticus, all Stenocereus thurberi within Organ Pipe Cactus National Monument), and provide a rationale for the sample choice. When relevant, describe the organism taxa, source, sex, age range and any manipulations. State what population the sample is meant to represent when applicable. For studies involving existing datasets, describe the data and its source.*
Sampling strategy	*Note the sampling procedure. Describe the statistical methods that were used to predetermine sample size OR if no sample-size calculation was performed, describe how sample sizes were chosen and provide a rationale for why these sample sizes are sufficient.*
Data collection	*Describe the data collection procedure, including who recorded the data and how.*
Timing and spatial scale	*Indicate the start and stop dates of data collection, noting the frequency and periodicity of sampling and providing a rationale for these choices. If there is a gap between collection periods, state the dates for each sample cohort. Specify the spatial scale from which the data are taken*
Data exclusions	*If no data were excluded from the analyses, state so OR if data were excluded, describe the exclusions and the rationale behind them, indicating whether exclusion criteria were pre-established.*
Reproducibility	*Describe the measures taken to verify the reproducibility of experimental findings. For each experiment, note whether any attempts to repeat the experiment failed OR state that all attempts to repeat the experiment were successful.*
Randomization	*Describe how samples/organisms/participants were allocated into groups. If allocation was not random, describe how covariates were controlled. If this is not relevant to your study, explain why.*
Blinding	*Describe the extent of blinding used during data acquisition and analysis. If blinding was not possible, describe why OR explain why blinding was not relevant to your study.*

Did the study involve field work? ☒ Yes ☐ No

Field work, collection and transport

Field conditions	Archaeological excavation in limestone cave
Location	East Kalimantan Indonesia, 1° 3'52.83"N 117°16'24.61"E
Access & import/export	All archaeological research and transport of materials is conducted in agreement with our collaborators and coauthors from Indonesian institutions: BRIN, OR Arkeologi, in Jakarta, as well as Balai Pelestarian Cagar Budaya Kalimantan Timur, Samarinda. Both institutions have supported and facilitated research permits for all Australian researchers involved in fieldwork.
Disturbance	Small sample of very large cave, most likely to be less than 1 percent of potential archaeological deposit at site.

Reporting for specific materials, systems and methods

We require information from authors about some types of materials, experimental systems and methods used in many studies. Here, indicate whether each material, system or method listed is relevant to your study. If you are not sure if a list item applies to your research, read the appropriate section before selecting a response.

Materials & experimental systems

n/a	Involved in the study
☒	☐ Antibodies
☒	☐ Eukaryotic cell lines
☐	☒ Palaeontology and archaeology
☒	☐ Animals and other organisms
☒	☐ Human research participants
☒	☐ Clinical data
☒	☐ Dual use research of concern

Methods

n/a	Involved in the study
☒	☐ ChIP-seq
☒	☐ Flow cytometry
☒	☐ MRI-based neuroimaging

Palaeontology and Archaeology

Specimen provenance

East Kalimantan Indonesia. Research permits obtained from Pusat Penelitian Arkeologi Nasional, authorised by Dr I Made Geria in Juny 2019.

Specimen deposition

Material currently housed at Griffith University, where Indonesian and Australian researchers have present access.

Dating methods

A total of 10 in situ radiocarbon dating charcoal samples were dated by (AMS14C) at the Direct AMS laboratory, in Seattle U.S.A. Dates are calibrated using OxCal v. 4.4, with the Northern Hemisphere Atmospheric curve [IntCal20]38. Samples were pre-treated with ABA protocols following 6M HCl, 65C 12 minutes followed by DI water rinse, 6M HCl 65C 12 minutes followed by 3 DI water rinses, 0.09M KOH 65C 12 minutes followed by DI water rinse, then 0.05M HCl rinse. This base step with subsequent rinses is repeated twice more. Finally, the pretreatment is finished with 2 additional 0.05M HCl rinses. D-AMS 038331 and 038334 received additional base step(s), for a total of 4 and 5 respectively. Sample D-AMS 038338 showed signs of breakdown in base and thus received a less aggressive ABA base step, following: 0.09M KOH, room temperature, 12 mins followed by DI water rinse and 0.05M HCl rinse, and 0.09M KOH, 65C, 12 mins followed by DI water rinse and finished with three 0.05 M HCl rinses.

A combined Uranium-series and Electron Spin Resonance (US-ESR) dating technique was undertaken on a sample of TB1's left mandibular molar (M3) and returned an age estimate of 25.4 ± 4.3 ka (1-sigma). This dating was undertaken at Southern Cross University at the GARG facility. The tooth was first cut in half using a rotating diamond saw with a blade of 300 microns, before being polished to 5 micron smoothness. The sample was then analysed for uranium-series isotopes and concentration in both dentine and enamel using a laser ablation NWR ESI 213 laser coupled with a MC-ICPMS Neptune XT from Thermo Fisher to calculate the internal dose rate. An enamel fragment was then measured on a Freiberg MS5000 ESR X-band spectrometer and irradiated with the Freiberg X-ray irradiation chamber. ESR intensities were extracted from the merged spectra obtained on the angular variation measurements, after correcting for baseline, subtraction of isotropic signals, and assessment of NOCORS contribution. Dose response curve were obtained using the MCDOSE 2.0 software42.

Incorporating the ESR age into the Bayesian model, provides a modelled age of 31,201 to 30,714 (2-sigma or 95.4% probability) for the reported burial.

☒ Tick this box to confirm that the raw and calibrated dates are available in the paper or in Supplementary Information.

Ethics oversight

Griffith University, Brisbane Australia; and BRIN Arkeologi, Bahasa dan Sastra, Pusat Riset Lingkungan, Maritim, dan Budaya Berkelanjutan, Jakarta, Indonesia.

Note that full information on the approval of the study protocol must also be provided in the manuscript.
